# Development of a composite diffusion tensor imaging score correlating with short-term neurological status in neonatal hypoxic–ischemic encephalopathy

**DOI:** 10.3389/fnins.2022.931360

**Published:** 2022-08-02

**Authors:** Kengo Onda, Eva Catenaccio, Jill Chotiyanonta, Raul Chavez-Valdez, Avner Meoded, Bruno P. Soares, Aylin Tekes, Harisa Spahic, Sarah C. Miller, Sarah-Jane Parker, Charlamaine Parkinson, Dhananjay M. Vaidya, Ernest M. Graham, Carl E. Stafstrom, Allen D. Everett, Frances J. Northington, Kenichi Oishi

**Affiliations:** ^1^The Russell H. Morgan Department of Radiology and Radiological Science, The Johns Hopkins University School of Medicine, Baltimore, MD, United States; ^2^Division of Pediatric Neurology, Department of Pediatrics, Children's Hospital of Philadelphia, Philadelphia, PA, United States; ^3^Neuroscience Intensive Care Nursery Program, Division of Neonatology, The Johns Hopkins University School of Medicine, Baltimore, MD, United States; ^4^Division of Neonatology, Department of Pediatrics, The Johns Hopkins University School of Medicine, Baltimore, MD, United States; ^5^Edward B. Singleton Department of Radiology, Texas Children's Hospital, Baylor College of Medicine, Houston, TX, United States; ^6^Division of Neuroradiology, Department of Radiology, Larner College of Medicine at the University of Vermont, Burlington, VT, United States; ^7^Division of Pediatric Radiology and Pediatric Neuroradiology, Department of Radiology, The Johns Hopkins University School of Medicine, Baltimore, MD, United States; ^8^St. George's University of London, London, United Kingdom; ^9^Department of General Internal Medicine, The Johns Hopkins University School of Medicine, Baltimore, MD, United States; ^10^Division of Maternal-Fetal Medicine, Department of Gynecology and Obstetrics, The Johns Hopkins University School of Medicine, Baltimore, MD, United States; ^11^Division of Pediatric Neurology, Department of Neurology, The Johns Hopkins University School of Medicine, Baltimore, MD, United States; ^12^Division of Pediatric Cardiology, Department of Pediatrics, The Johns Hopkins University School of Medicine, Baltimore, MD, United States

**Keywords:** hypoxic-ischemic encephalopathy, outcome prediction, diffusion tensor imaging, neonatal brain atlas, least absolute shrinkage and selection operator, short-term neurologic outcome, serum biomarkers

## Abstract

Hypoxic–ischemic encephalopathy (HIE) is the most common cause of neonatal acquired brain injury. Although conventional MRI may predict neurodevelopmental outcomes, accurate prognostication remains difficult. As diffusion tensor imaging (DTI) may provide an additional diagnostic and prognostic value over conventional MRI, we aimed to develop a composite DTI (cDTI) score to relate to short-term neurological function. Sixty prospective neonates treated with therapeutic hypothermia (TH) for HIE were evaluated with DTI, with a voxel size of 1 × 1 × 2 mm. Fractional anisotropy (FA) and mean diffusivity (MD) from 100 neuroanatomical regions (FA/MD ^*^100 = 200 DTI parameters in total) were quantified using an atlas-based image parcellation technique. A least absolute shrinkage and selection operator (LASSO) regression was applied to the DTI parameters to generate the cDTI score. Time to full oral nutrition [short-term oral feeding (STO) score] was used as a measure of short-term neurological function and was correlated with extracted DTI features. Seventeen DTI parameters were selected with LASSO and built into the final unbiased regression model. The selected factors included FA or MD values of the limbic structures, the corticospinal tract, and the frontotemporal cortices. While the cDTI score strongly correlated with the STO score (rho = 0.83, *p* = 2.8 × 10^−16^), it only weakly correlated with the Sarnat score (rho = 0.27, *p* = 0.035) and moderately with the NICHD-NRN neuroimaging score (rho = 0.43, *p* = 6.6 × 10^−04^). In contrast to the cDTI score, the NICHD-NRN score only moderately correlated with the STO score (rho = 0.37, *p* = 0.0037). Using a mixed-model analysis, interleukin-10 at admission to the NICU (p = 1.5 × 10^−13^) and tau protein at the end of TH/rewarming (*p* = 0.036) and after rewarming (*p* = 0.0015) were significantly associated with higher cDTI scores, suggesting that high cDTI scores were related to the intensity of the early inflammatory response and the severity of neuronal impairment after TH. In conclusion, a data-driven unbiased approach was applied to identify anatomical structures associated with some aspects of neurological function of HIE neonates after cooling and to build a cDTI score, which was correlated with the severity of short-term neurological functions.

## Introduction

Neonatal hypoxic–ischemic encephalopathy (HIE) is the most common neonatal acquired brain injury and is caused by the disruption of cerebral blood flow and oxygen supply near birth. HIE can lead to significant lifelong neurological morbidity (Douglas-Escobar and Weiss, 2015), and HIE represents about half of all cases of neonatal encephalopathy. Therapeutic hypothermia (TH) reduces by one-third the death and major disability in neonates with moderate-to-severe HIE (Gluckman et al., 2005; Shankaran et al., 2005; Tagin et al., 2012). Identifying which neonates are at the highest risk of poor neurological outcomes despite TH is still difficult, and accurate prognostic indicators are needed.

Assessment of injury by qualitative and quantitative analyses of magnetic resonance imaging (MRI) has been correlated with short- and long-term outcomes in HIE (van Laerhoven et al., 2013; Massaro, 2015; Shankaran et al., 2015). However, conventional MRI may under-estimate injury, and advanced techniques, including diffusion tensor imaging (DTI), provide an additional diagnostic and prognostic value (Thayyil et al., 2010; Alderliesten et al., 2011; Martinez-Biarge et al., 2011; van Laerhoven et al., 2013) by detecting mild neuronal injury that is difficult to evaluate with conventional MRI sequences. Prior research using DTI data has been limited by the need for the manual segmentation of regions of interest (ROIs), which are labor-intensive and require anatomical expertise, thus limiting both the number of patients and the number of regions that can be evaluated (Massaro et al., 2015; Lemmon et al., 2017; Seo et al., 2017; Jang and Kwon, 2018; Gerner et al., 2019; Salas et al., 2019; Longo et al., 2020). Moreover, many of the previously published studies have relied on the hypothesis-driven identification of brain regions known to be involved in HIE, including brainstem, basal ganglia, thalamus, posterior limb of the internal capsule, postcentral gyrus, and cortical white matter. Voxel-based analysis using tract-based spatial statistics (TBSS) is a data-driven approach, but is limited to white matter tracts (Tusor et al., 2012; Ly et al., 2015). Moreover, although previous studies have shown a relationship between anatomical impairment in specific brain regions and clinical severity, the prediction of prognosis has remained problematic, especially for mild cases (Zarifi et al., 2002; Rollins et al., 2014; Bano et al., 2017), suggesting the need to introduce a predictive model that combines DTI findings from multiple areas of the brain in an unbiased manner.

To overcome the limitations of previous studies, we used an atlas-based approach to parcellate whole-brain DTI into 122 anatomical regions covering the whole brain, including both gray and white matter structures (Oishi et al., 2011). The DTI information in these regions was analyzed by the LASSO regression analysis to create a model that outputs a numerical value [composite DTI (cDTI) score] correlating with time to full oral feedings as a short-term neuro-functional measure [short-term oral feeding (STO) score]. Serum biomarkers previously reported in this cohort of HIE newborns (Dietrick et al., 2020; Chavez-Valdez et al., 2021b) were used to elucidate the longitudinal mechanistic origins related to the cDTI score.

## Materials and methods

### Participants

Data were obtained from a prospective cohort of neonates who underwent TH for neonatal encephalopathy at the Johns Hopkins Hospital, Baltimore, MD, USA. The study was approved by the institutional review board. A diagnosis of HIE was based on the National Institute of Child Health and Human Development (NICHD) Neonatal Research Network criteria (Shankaran et al., 2005). Out of 659 cases in total, 535 cases were excluded due to a lack of brain MRI. In addition, neonates with arterial ischemic stroke or IVH or both (*n* = 21), neonates requiring extracorporeal membrane oxygenation (ECMO) (*n* = 4), non-perinatal events (*n* = 3), incomplete clinical data (*n* = 3), partial TH administration (<72 h) (*n* = 2), TH off-label use (<35-week gestation at birth) (*n* = 1), and non-HIE neonatal encephalopathy (*n* = 1) were also excluded; three of the 89 eligible neonates were excluded due to motion artifact from the baby's arousal during scanning. Additionally, DTIs that did not meet the voxel size criteria (*n* = 22) and neonates who were older than 14 days at the time of the MRI scan (*n* = 4) were excluded. A total of 60 patients were included in the final analysis.

### Clinical variables

**Figure 1:**
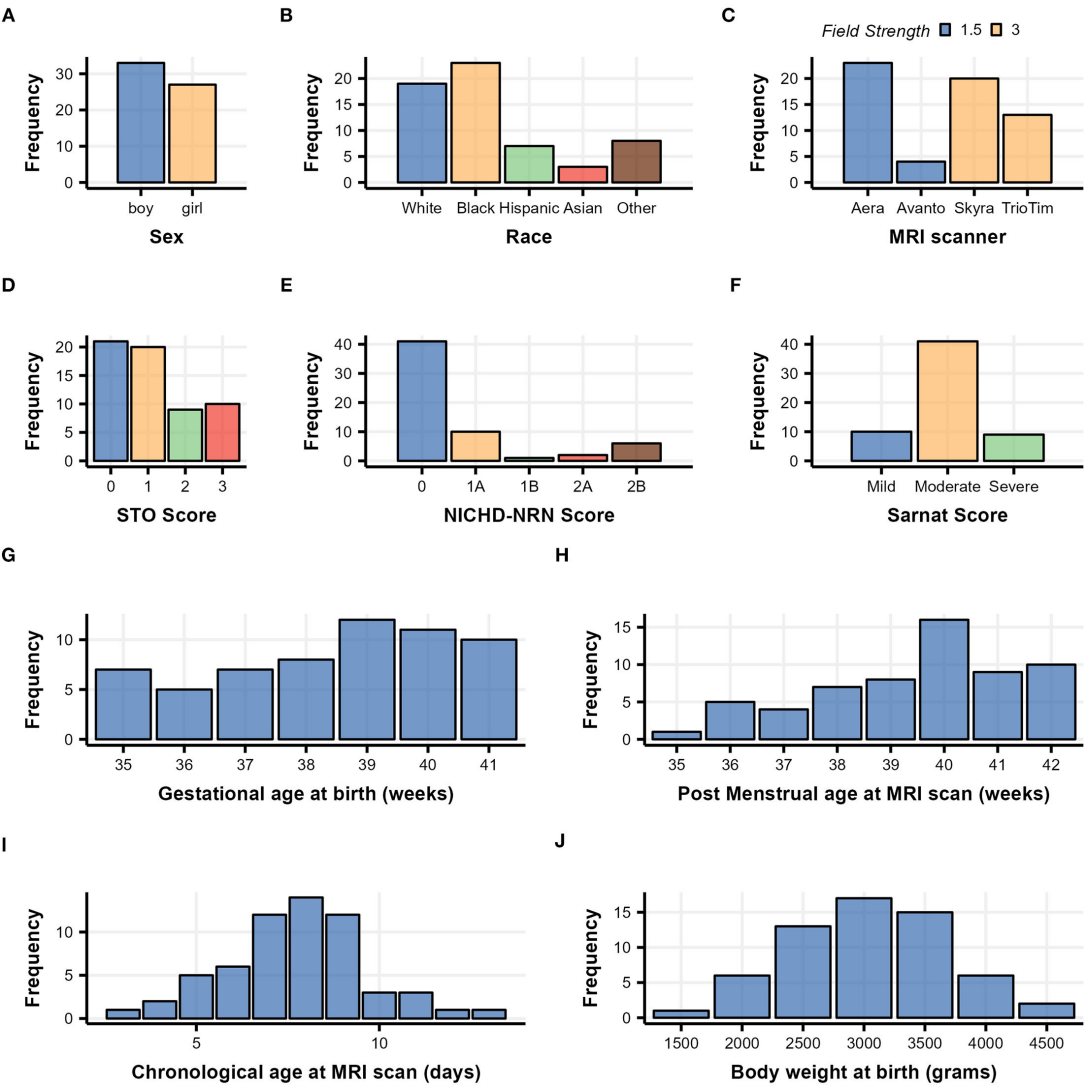
Demographic histograms of 60 subjects with neonatal HIE who underwent therapeutic hypothermia treatment. Categorical clinical variables are summarized in **(A–F)** with color scaled by variables, and continuous clinical variables are summarized in **(G–J)**.

Clinical data were obtained from the medical record. The race was assigned based on maternal race. The sex and GA were assigned by the NICU team at admission to the NICU. The highest modified Sarnat score during the first 6 h of admission to the NICU was determined by the study team (RC-V, CP, and FJN) (Sarnat and Sarnat, 1976). The distributions of clinical categorical/numerical characteristics, such as sex, race, NICHD-NRN score, Sarnat score, GA at birth, and post-menstrual age (PMA) at MRI scan, chronological age at MRI scan, and birth weight (BW) are summarized in Figure 1 and Table 1.

**Table 1:** Clinical characteristics of 60 neonatal HIE patients who underwent therapeutic hypothermia treatment.

**Categorical clinical variables, n / N (%)**	***N* = 60**
**Sex of patients**	
Male	33 (55%)
Female	27 (45%)
**Race**	
White	19 (32%)
Black	23 (38%)
Hispanic	7 (12%)
Asian	3 (5.0%)
other	8 (13%)
**STO score**	
0	21 (35%)
1	20 (33%)
2	9 (15%)
3	10 (17%)
**Sarnat score**	
Mild	10 (17%)
Moderate	41 (68%)
Severe	9 (15%)
**NICHD-NRN score**	
0	41 (68%)
1A	10 (17%)
1B	1 (1.7%)
2A	2 (3.3%)
2B	6 (10%)
**Scanner type**	
Aera (1.5T)	23 (38%)
Avanto (1.5T)	4 (6.7%)
Skyra (3T)	20 (33%)
TrioTim (3T)	13 (22%)
**Continuous clinical variables, Mean (SD)**	***N*** **=** **60**
Gestational age at birth (weeks)	38.81 (1.92)
Post-menstrual age at MRI scan (weeks)	39.91 (1.89)
Chronological age at MRI scan (days)	7.73 (1.96)
Body weight at birth (grams)	3,299 (621)

### Short-term oral-feeding (STO) score

The STO score was designed as a measure of short-term neurological function, with a focus on the attainment of oral feeding (Graham et al., 2008; Badran et al., 2020). This score ranged from 0 to 4. For all neonates undergoing TH, no feeds were offered until the day of life (DOL) 3, which corresponded to the end of the rewarming phase. Thus, a score of 0 was assigned if a patient achieved full oral feeds within 3 days after initiation ( ≤ 7 days of life); a score of 1 was assigned if 3 extra days were needed (8–10 days of life), which corresponded to the standard weaning of parenteral fluids and transition to enteral feeds; a score of 2 was assigned if a patient achieved full oral feeding in ≤ 5 weeks; a score of 3 was assigned if > 5 weeks was needed or a gastrostomy tube (G-tube) was placed for discharge; and a score of 4 was assigned if a patient died during the hospitalization due to withdrawal of care as a result of the severity of brain injury. Data were obtained by RC-V, HS, SM, and CP, and the data were revised and scored by RC-V.

### Serum biomarkers

Serum levels of central nervous system injury (glial fibrillary acidic protein [GFAP], neurogranin [NRGN], tau), inflammation (interleukin [IL]-6, IL-8, IL-10), and trophism (brain-derived neurotrophic factor [BDNF] and vascular endothelial growth factor [VEGF]) proteins were available for those patients included in the study. Serial samples at up to eight separate time periods (from DOL 0 to 7) were measured from stored laboratory samples using a custom, multiplex assay (Meso Scale Discovery [MSD], Rockville, MD, USA) as previously described (Dietrick et al., 2020; Chavez-Valdez et al., 2021b). For each patient, the measurements were reorganized into four time points, namely, baseline [admission to NICU], during TH, end of TH/rewarming, and after rewarming, according to the DOL; the value sampled at DOL 0 was set as baseline; the larger value sampled at DOL 1 and 2 as during TH; the larger value sampled at DOL 3 and 4 as the end of TH/rewarming; and the largest value sampled at DOL 5, 6, and 7 as after rewarming.

### MRI acquisition

The MR imaging studies were acquired at either 1.5 tesla or 3.0 tesla on four clinical types of MR scanners, namely, Aera, Avanto, Skyra, and TrioTim (Siemens, Erlangen, Germany), using a standard eight-channel head coil. The neonatal imaging protocols included a single-shot spin-echo, echo-planar axial DTI sequence with diffusion gradients along 20 noncollinear directions. For each of the 20 diffusion-encoding directions, a *b*-value of 800 s/mm^2^ was used for four patients and 1,000 s/mm^2^ was used for the rest of the patients. An additional measurement without diffusion weighting (b0 s/mm^2^) was taken. The voxel size was 1 × 1 × 2 mm. The distributions of field strength, field of view (FOV), and *b*-value among the four types of MR scanners are summarized in Figure 1C and Table 2.

**Table 2:** MRI scan parameters used in this study.

**Scanner type, n/N (%)**	**Aera, *N* = 23**	**Avanto, *N* = 4**	**Skyra, *N* = 20**	**TrioTim, *N* = 13**
**FOV**				
1,344 ×1,344	0 (0%)	0 (0%)	1 (5.0%)	0 (0%)
1,536 ×1,536	23 (100%)	4 (100%)	19 (95%)	9 (69%)
1,728 ×1,728	0 (0%)	0 (0%)	0 (0%)	4 (31%)
**Field strength**				
	1.5T	1.5T	3T	3T
***B*** **-value**				
800 s/mm^2^	0 (0%)	0 (0%)	0 (0%)	4 (31%)
1,000 s/mm^2^	23 (100%)	4 (100%)	20 (100%)	9 (69%)

### Evaluation of the impact of differences in MR scanner, magnetic field strength, and *b*-value on DTI quantification

As the four different scanners, namely, Aera, Avanto, Skyra, and TrioTim, were used to obtain DTI for this population, we first investigated whether the neurological severity of the neonates was evenly distributed among the scanners used. We built a proportional odds model and performed a type II likelihood ratio test on the model to evaluate the difference in the STO score distribution among the four MR scanners. To compare the effect size of the MR scanners, the preference of the STO score was estimated based on the model for each MR scanner, and those estimations were analyzed through the type II likelihood ratio test. The Brant–Wald test was conducted to check whether there was a violation of the proportional odds model assumption. We used the MASS library version 7.3-54 for model building and the Brant library version 0.3-0 for testing, both of which run on R (version 4.1.2). The same analysis was performed for two field strength levels (1.5T/3.0T), and the results are summarized in Supplementary Table 1. Then, we examined the impact of the scanner used on the cDTI score, applying Spearman's rank pairwise correlation test to investigate the correlation between the cDTI score and the use of each scanner (not used = 0 and used = 1), as described in Section “Relationship between the cDTI score and the clinical variables.” To determine the effect of including different *b*-values (four patients were scanned with *b* = 800 s/mm^2^ and the rest of the patients were scanned with 1,000 s/mm^2^) on the results, an additional analysis was performed on a group of 56 patients using a *b*-value of 1,000 s/mm^2^.

### Clinical scoring of the T1- and T2-weighted images and diffusion-weighted images

Two experienced pediatric neuroradiologists (BPS and AT) scored the neonatal brain MRIs using the National Institute of Child Health and Human Development (NICHD) Neonatal Research Network (NRN) score (Shankaran et al., 2015). In addition, the overall image quality of all sequences was reviewed to determine the quality of DTI data. Throughout the quality control, three MRIs that had significant artifacts were excluded from the subsequent quantitative DTI analysis, as described in Section “Participant.”

### Atlas-based image analysis

**Figure 2:**
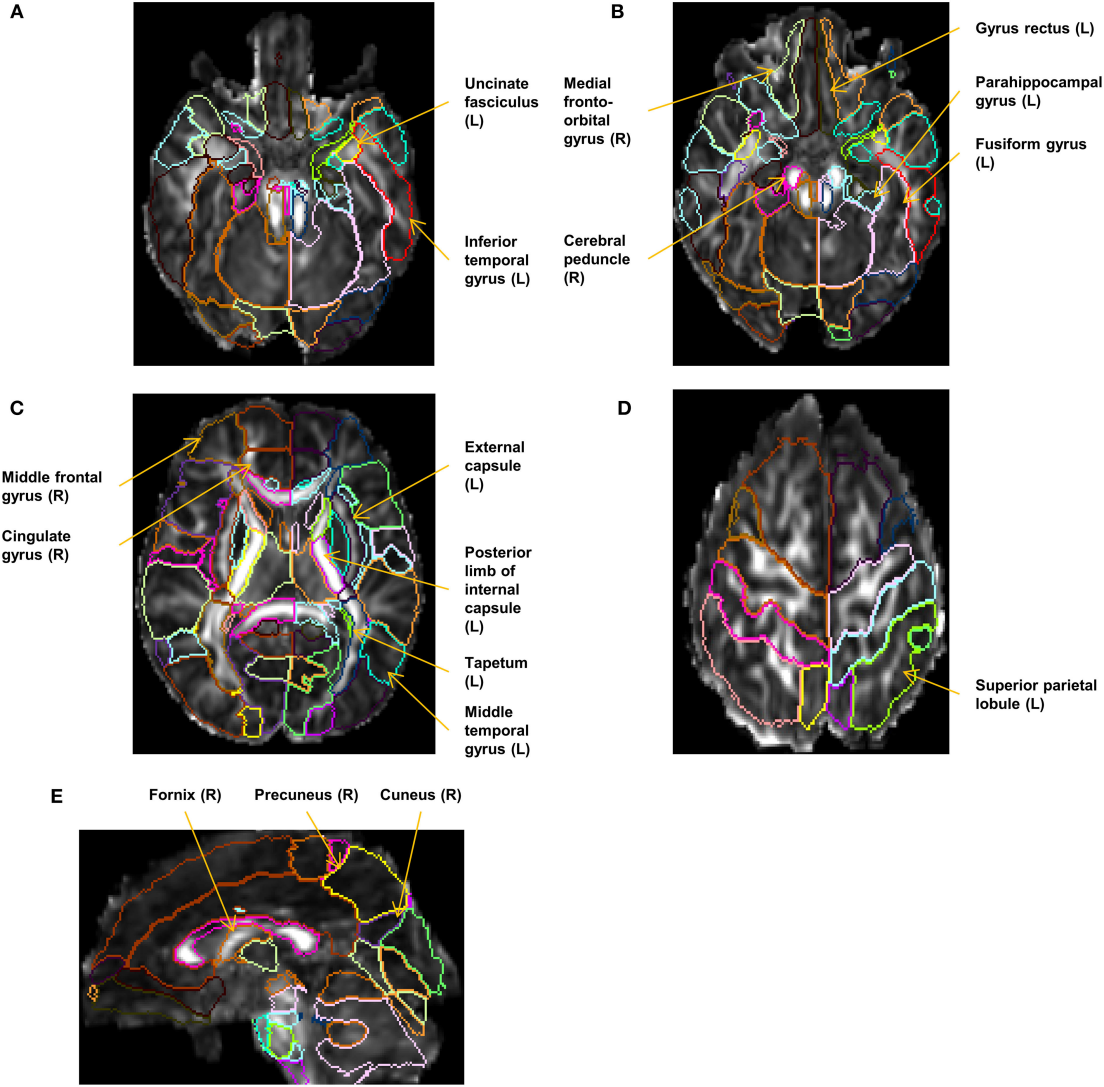
Representative parcellation maps superimposed on FA maps. The locations and laterality (L/R) of the selected 17 structures in the cDTI score calculation are annotated. **(A–D)** Axial images at the level of the corticospinal tract, the uncinate fasciculus **(A)**, the cerebral peduncle **(B)**, the basal ganglia **(C)**, and the superior parietal lobule **(D)**. **(E)** A sagittal image at the level of the right fornix and the right cuneus.

The diffusion-weighted images were first linearly registered to the b0 image, followed by voxel-wise tensor fitting using DtiStudio (www.mristudio.org) (Jiang et al., 2006). An automated outlier rejection function (Li et al., 2013) was applied to reject slices with a relative fitting error of more than 3%. The fractional anisotropy (FA) and the mean diffusivity (MD) maps were calculated from the tensor field. The JHU-neonate single-brain DTI atlas and the parcellation map that contains 122 anatomical areas as the regions of interest (ROIs) (Oishi et al., 2011) were transformed into each individual's FA and MD images through the dual-channel (FA and MD) large deformation diffeomorphic metric mapping (LDDMM), as described in Oishi et al. (2011), Akazawa et al. (2016), and Wu et al. (2017a,b). Among 122 ROIs defined on each neonate's brain, 100 ROIs with a minimum volume greater than 2 mm^3^ were analyzed as reliable ROIs (Otsuka et al., 2019). The names of the 100 ROIs are provided in Supplementary Table 2. Two of the authors (KOn. and KOi) inspected the resultant parcellation maps and identified eight parcellation maps with minor errors in structural boundaries, which were manually corrected. For each ROI, an FA threshold of > 0.2 was applied to select white matter areas and fiber-rich components within the deep gray matter. The mean FA and MD values were quantified for statistical analysis. Figure 2 shows the representative parcellation maps overlaid on FA maps.

### LASSO regression model

The least absolute shrinkage and selection operator (LASSO) regression method with cross-validation was applied to extract important DTI features from 200 measures (100 ROIs × 2 measures [FA, MD]) and generate a cDTI score for neonatal HIE that correlated with the STO score for each individual. The LASSO regression model was chosen as a sparse model that addresses the overfitting and multicollinearity problem expected in whole-brain DTI analysis and includes only variables that have a significant impact on the STO scores. The DTI measures were converted to a z-score based on the mean and the standard deviation of each DTI-derived measurement of each ROI and served as input variables. The best lambda parameter was defined by 10-fold cross-validation, setting the alpha parameter as 1, to minimize a mean squared prediction error between the measured and the predicted STO scores. A software package, glmnet version 4.1-3, that runs on R (version 4.1.2) was used for the analysis (Friedman et al., 2010). Spearman's rank correlation tests with the cDTI score were performed for all factors selected in the prediction model, and the results are presented in scatterplots with regression lines and coefficients (Supplementary Figure 1).

### Relationship between the cDTI score and the clinical variables

The Spearman's rank pairwise correlation test was used to evaluate the relationship between the categorical variables (i.e., STO score, Sarnat score, NICHD-NRN score, sex, field strength, with or without the use of each MR scanner) and the Pearson's pairwise correlation test was used to evaluate the relationship between the numerical variables (i.e., GA and BW at birth, and chronological age and PMA at MRI scan), both including the cDTI score. A *p* < 0.05 was regarded as a significant correlation.

### Comparison of biomarkers between severe and mild groups over time

Based on the cDTI score, all patients were classified into mild or severe groups. A cDTI score of 1.5 or less was defined as the mild group and above 1.5 as the severe group. The cut point of cDTI score of 1.5 was set to separate neonates with mild MRI findings from those with typical lesions in the basal ganglia–thalamus, watershed area, and the internal capsule, with approximately the same number of subjects in the mild and severe groups. Using lme4 library version 1.1-27.1 on R (version 4.1.2), a linear mixed-effects analysis was performed for each biomarker (i.e., BDNF, IL-6, VEGF, GFAP, NRGN, IL-10, IL-8, and tau). The concentration values of biomarkers were set as a response, and the dichotomized severity (mild/severe), the four time points (i.e., baseline, during TH, end of TH/rewarming, and after rewarming), and the interaction of both were set as fixed effects, and the intercepts for subjects were set as random effects. A type II Wald *F*-test with a Kenward–Roger degree of freedom was conducted on each mixed model to see the overall difference in biomarker values among the four different time points, two levels of severity, and their interaction.

For biomarkers in which significant differences were identified between the mild and severe groups, Welch's *t*-test was further performed as a *post-hoc* test using the emmeans library version 1.7.2 on R (version 4.1.2) to identify at which time points there were differences. Furthermore, the correlation between the cDTI score and biomarker values at each time point was examined using Spearman's rank correlation method. For all series of analyses (i.e., *F*-test, *post-hoc* tests, and correlation test), the significance level of the *p*-value was set at 0.05.

## Results

### Participants and distribution of clinical variables

Sixty patients met our clinical inclusion criteria and had high-quality DTI available for quantitative analysis. Overall, the group tended to have mild-to-moderate rather than moderate-to-severe injury, as evidenced by the distribution of the Sarnat scores and NICHD-NRN scores shown in Figures 1E,F and Table 1. The chronological age at the MRI scan averaged 7.7 days, indicating that the DTI captured the subacute phase of HIE when a diffusion-weighted image is most informative in determining the overall extent of injury (Huang and Castillo, 2008; Ouwehand et al., 2020).

### The effect of different MR scanners and field strength levels

The result of the type II likelihood ratio test on the proportional odds model showed no significant differences in the STO score distribution among four MR scanner types (*p* = 0.73) or between two levels of field strength (*p* = 0.38), as described in Table 3 and Supplementary Table 1. The Brant–Wald test was performed to assess the validity of the proportional odds model used for both tests. No significant *p*-values were found for any items, indicating that the proportional odds model assumption was not violated, and the results of the proportional odds model were valid. Thus, all 60 MR data were pooled together for the subsequent cDTI score calculation.

**Table 3:** Results of the proportional odds model analysis for the evaluation of the distribution of the STO score among four MR scanners.

**Analysis of deviance table (Type II test)**			
**Variable**	**χ^2^**	**df**	***p*** **-value**
MRI scanner	1.3	3	0.73
**Brant test table for the proportional odds model**			
**Variable tested for**	**χ^2^**	**df**	***p*** **-value** ^**a**^
Aera	0.18	2	0.91
Avanto	2.3	2	0.32
Skyra	0.19	2	0.91
TrioTim	10	6	0.11

^a^If there are no significant p-values, the model satisfies the proportional odds assumption.

### Generation of the cDTI score

Seventeen factors (FA of 10 ROIs and MD of 7 ROIs) were selected from 200 factors by the LASSO analysis and built into the final regression model. In this model, each variable (FA or MD derived from the 17 selected anatomical structures) was weighted and the sum (cDTI score) was calculated for each patient. The standardized regression coefficients for each of the image and non-image factors are presented in Table 4. Most of the 17 selected structures can be categorized as limbic fibers and related structures (parahippocampal gyrus, fornix, medial fronto-orbital gyrus, uncinate fasciculus, and cingulate gyrus); lateral frontotemporal cortices and related fibers (middle frontal gyrus, middle temporal gyrus, inferior temporal gyrus, and tapetum); and corticospinal projection fibers (cerebral peduncle and posterior limb of internal capsule). The locations and laterality of the selected factors are illustrated in Figure 2.

**Table 4:** Regression coefficients of the 17 factors selected by the LASSO regression analysis.

**Regression coefficient**	**DTI**	**Anatomical structure**	**Side**
**Positive regression coefficients**			
0.40	MD	Cuneus*	Right
0.064	MD	Parahippocampal gyrus*†	Right
**Negative regression coefficients**			
−0.31	MD	Middle frontal gyrus	Right
−0.23	FA	Fornix*†	Right
−0.22	MD	Inferior temporal gyrus	Left
−0.15	MD	Tapetum	Left
−0.13	FA	Medial fronto-orbital gyrus*†	Right
−0.12	FA	Uncinate fasciculus*†	Left
−0.072	FA	Fusiform gyrus*	Left
−0.068	FA	Gyrus rectus*	Left
−0.059	MD	Cerebral peduncle	Right
−0.057	FA	Precuneus	Right
−0.037	FA	External capsule*	Left
−0.031	FA	Superior parietal lobule*	Left
−0.017	FA	Middle temporal gyrus*	Left
−0.011	FA	Posterior limb of internal capsule	Left
−0.0091	MD	Cingulate gyrus†	Right

^*^Significant correlation with the cDTI score (Spearman's rank correlation, p < 0.05).

†Limbic fibers and related structures.

For the majority of the selected factors, increased MD and decreased FA were associated with higher cDTI scores, that is, worse neurological functions. On the contrary, a decrease in MD was shown to worsen the functions in five anatomical structures, including the lateral frontotemporal structures and the cerebral peduncle. Ten factors demonstrated moderate correlation with the cDTI score: cuneus MD (rho = 0.45); parahippocampal gyrus MD (rho = 0.32); middle temporal gyrus FA (rho = −0.28); superior parietal lobule FA (rho = −0.36); external capsule FA (rho = −0.34); gyrus rectus FA (rho = −0.40); fusiform gyrus FA (rho = −0.32); uncinate fasciculus FA (rho = −0.29); medial fronto-orbital gyrus FA (rho = −0.45); and fornix FA (rho = −0.59).

### Relationship between the cDTI score and the clinical variables

#### Severity scale comparison (STO/cDTI/NICHD-NRN)

**Figure 3:**
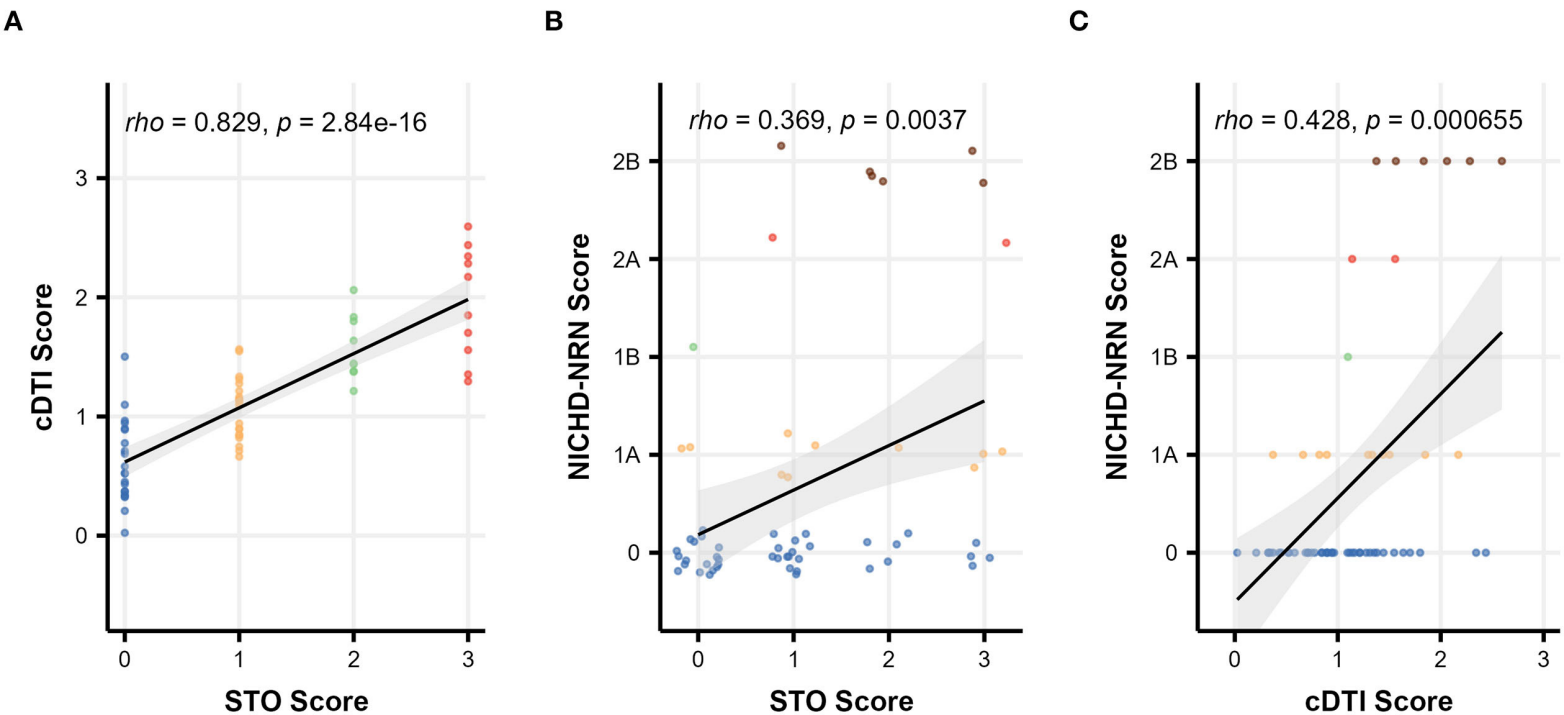
Scatterplots showing the relationship among severity scales: comparison between **(A)** the cDTI score and the STO score, **(B)** the NICHD-NRN score and the STO score, and **(C)** the NICHD-NRN score and the cDTI score. Solid black lines with gray areas represent the regression lines with 95% confidence intervals, and Spearman's correlation coefficients/*p*-values are shown in the upper left corner of each graph. For **(B)**, the data are jittered to show the sample size.

Figure 3 illustrates the relationship between three severity scales, namely, STO, cDTI, and NICHD-NRN. An excellent correlation was achieved between the STO score and the cDTI score, with a Spearman's rank correlation rho of 0.83 (*p* = 2.8 × 10^−16^, Figure 3A). For comparison, a correlation between the STO score and the NICHD-NRN score was also evaluated. The correlation (rho = 0.37, *p* = 0.0037) was significant, but weaker than the one between the STO and the cDTI scores (Figure 3B). As shown in the scatterplots in Figures 3B,C, higher NICHD-NRN and cDTI scores were associated with worse neurological functions. However, lower NICHD-NRN scores (e.g., 0, 1A, or 1B) were not necessarily associated with better neurological functions, whereas lower cDTI scores were associated with better neurological functions as represented by lower STO scores. This result suggested that the cDTI score is highly sensitive in detecting neuroanatomical alterations that are difficult to identify with conventional MRI sequences.

#### Relationships between cDTI score and demographics, clinical variables, and scanners

**Figure 4:**
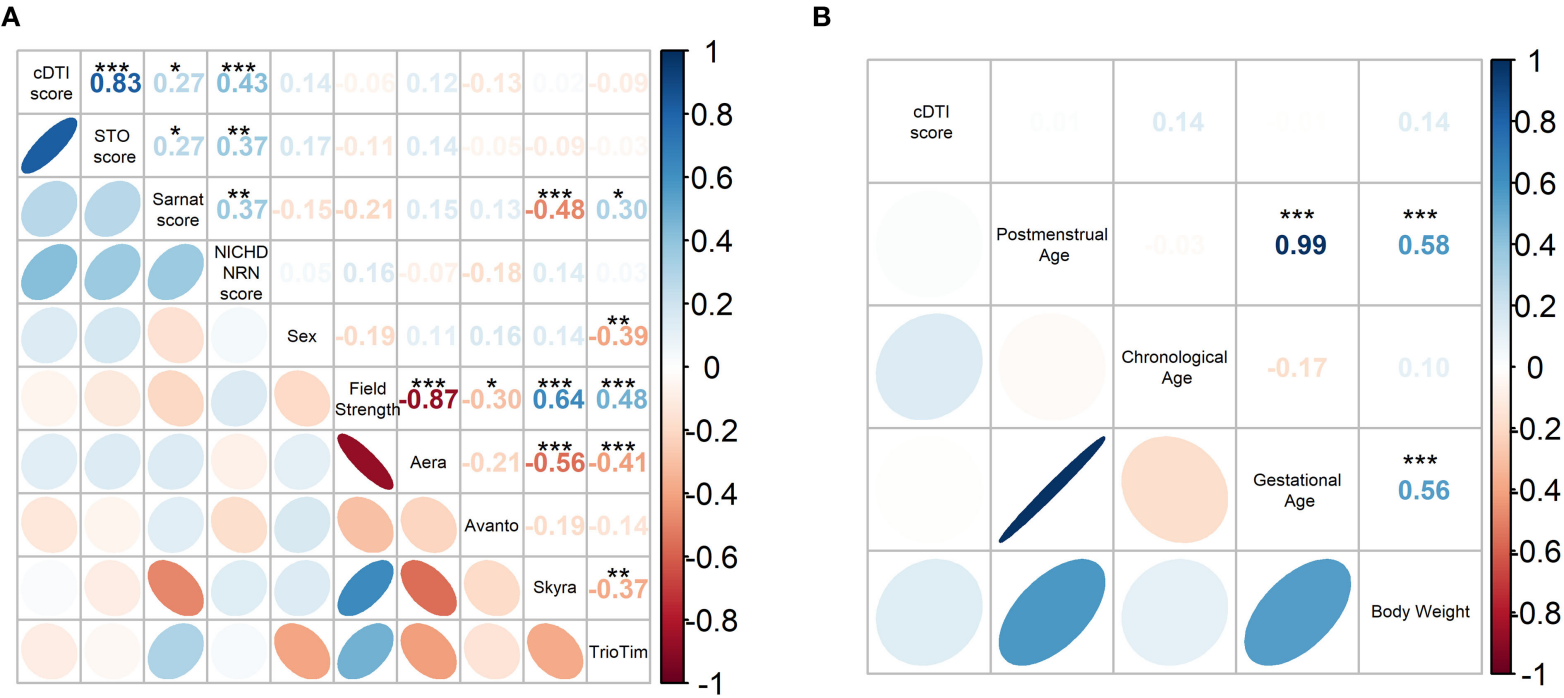
**(A)** Spearman's correlation coefficient matrix between the cDTI score and categorical clinical variables (i.e., STO, Sarnat, NICHD-NRN, sex, field strength, scanner preference). **(B)** Pearson's correlation coefficient matrix between the cDTI scores and numerical variables (postmenstrual age, chronological age, gestational age, and weight). Color-coded numbers in the upper right half of the matrix indicate correlation coefficients (*p < 0.05, **p < 0.01, and ***p < 0.001, blue: positive coefficient, red: negative coefficient). The color-coded ellipses in the lower left half of the matrix indicate the strength of correlation between variables, with blue indicating a negative correlation and red indicating a positive correlation. The shape of the ellipses indicates the strength of the correlation (ellipses are sharp when the correlation is strong and round when it is weak), positive slope indicates a positive correlation, and negative slope indicates a negative correlation.

The pairwise Spearman's correlation test revealed that the cDTI score had a strong correlation with the STO score and had a weak or a moderate correlation with the Sarnat score (rho = 0.27, *p* = 0.035) and the NICHD-NRN score (rho = 0.43, *p* = 6.6 × 10^−4^) (Figure 4A and Table 5). Correlations between the cDTI scores and sex, field strength, MR scanner preference, and all numerical clinical variables were not significant, as summarized in Figure 4 and Tables 5, 6.

**Table 5:** Spearman's correlation coefficients and *p*-values between the cDTI score and the categorical clinical variables.

**Statistic**	**STO** **score**	**Sarnat** **score**	**NICHD** **NRN** **score**	**Sex**	**Field** **strength**	**Aera**	**Avanto**	**Skyra**	**TrioTim**
rho	**0.83**	**0.27**	**0.43**	0.14	−0.057	0.12	−0.13	0.023	−0.095
*p*-value^a^	**2.8** **×10**^−16^***	0.035*	**6.6** **×10**^**−**4^***	0.28	0.66	0.35	0.33	0.87	0.47

^a^*p < 0.05 and ***p < 0.001. Correlation coefficients (rho) and p-values in bold indicate significant correlations.

**Table 6:** Pearson's correlation coefficients and *p*-values between the cDTI score and the continuous clinical variables.

**Statistic**	**Postmenstrual age at MRI scan (weeks)**	**Chronological age at MRI scan (days)**	**Gestational age at birth (weeks)**	**Body weight at birth (grams)**
r	0.012	0.14	−0.0088	0.14
*p*-value	0.93	0.29	0.95	0.30

### Comparison of biomarkers between severe and mild groups over time

A summary of the mixed-model analysis is presented in Table 7. The results of the *F*-test for the model indicated that there was a significant difference in biomarker values between the cDTI mild and severe groups for IL-10 (*p* = 4.1 × 10^−4^) and tau (*p* = 0.014). Significant time effects were present for BDNF (*p* = 0.0064), IL-6 (*p* = 0.030), VEGF (*p* = 6.1 × 10^−4^), GFAP (*p* = 0.016), and IL-10 (*p* = 5.9 × 10^−10^). Significant interactions between time point and severity were found for VEGF (*p* = 0.0043) and IL-10 (*p* = 1.7 × 10^−9^).

**Table 7:** Results of mixed-model analysis for each biomarker.

**Biomarker^a^**	**Source^b^**	**df**	**df (residual)**	***F*-value**	***p*-value^c^**
BDNF	**Timepoint**	3	105	4.3	0.0064**
	Severity	1	50	0.41	0.53
	Timepoint * Severity	3	105	0.76	0.52
IL-6	**Timepoint**	3	112	3.1	0.030*
	Severity	1	48	0.0073	0.93
	Timepoint * Severity	3	111	0.41	0.75
VEGF	**Timepoint**	3	99	6.3	0.00061***
	Severity	1	51	2.9	0.096
	**Timepoint** *Severity	3	100	4.7	0.0043**
GFAP	**Timepoint**	3	103	3.6	0.016*
	Severity	1	51	0.35	0.56
	Timepoint * Severity	3	103	0.11	0.96
NRGN	Timepoint	3	104	1.3	0.30
	Severity	1	50	0.35	0.56
	Timepoint * Severity	3	104	0.47	0.71
**IL-10**	**Timepoint**	3	95	20	**5.9** **×10**^−10^***
	**Severity**	1	35	15	0.00041***
	**Timepoint** *Severity	3	94	18	**1.7** **×10**^−9^***
IL-8	Timepoint	3	87	1.9	0.13
	Severity	1	39	0.30	0.59
	Timepoint * Severity	3	85	0.17	0.92
**Tau**	Timepoint	3	100	1.2	0.33
	**Severity**	1	46	6.6	0.014*
	Timepoint * Severity	3	100	2.0	0.12

^a^Biomarkers with significant differences between the severe and mild groups are made bold.

Severity is a binary variable of severity grouping (mild/severe) based on the cDTI score.

^b^Timepoint * Severity cells shows interactions of timepoint and severity variables.

^c^*p < 0.05, **p < 0.01, and ***p < 0.001.

**Figure 5:**
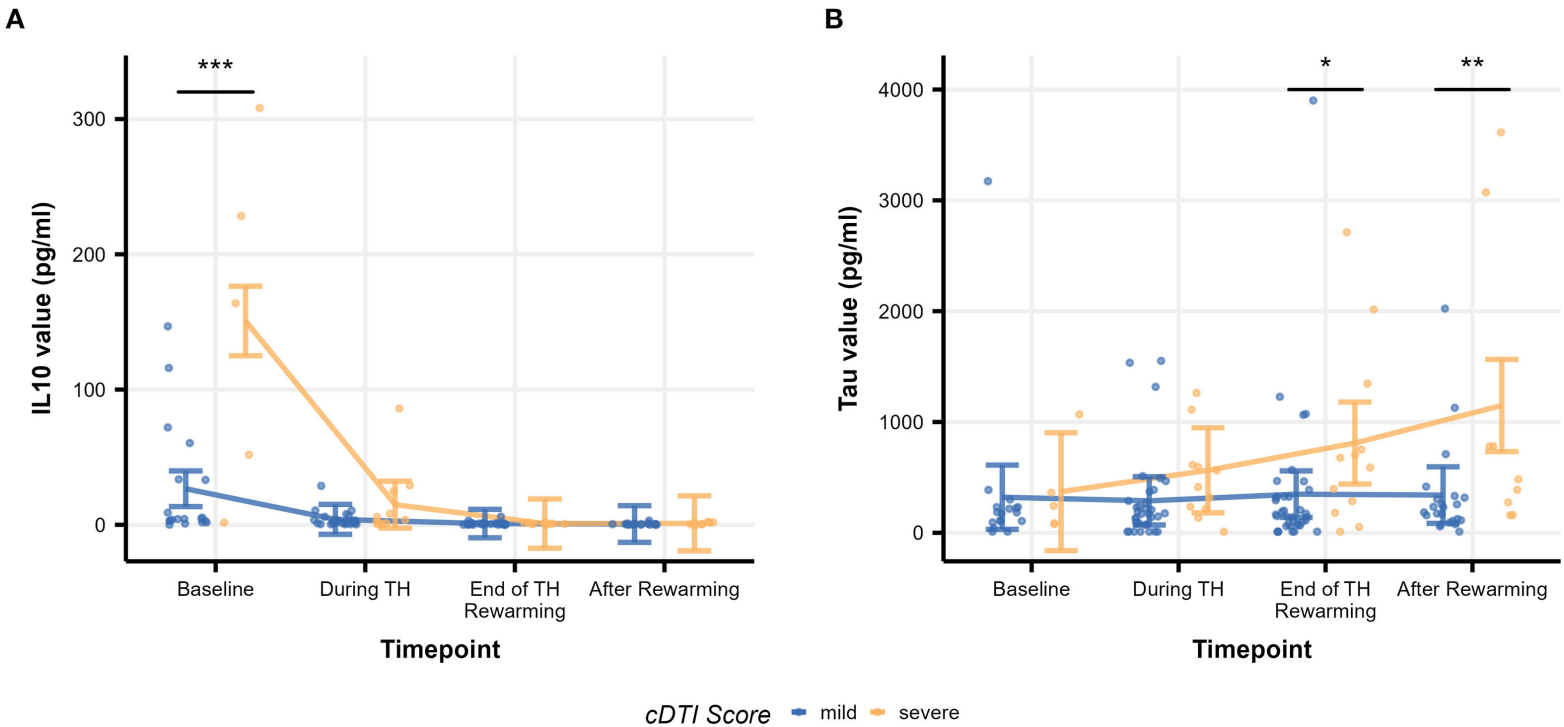
Time courses of biomarker values [**(A)**: IL-10 and **(B)**: tau] by severity group. Raw biomarker values are shown as scatterplots, and time courses are indicated as error bars. The error bars on each timepoint (baseline, during TH, end of TH/rewarming, and after rewarming) were calculated based on the results of the mixed-model analysis. Significance stars are embedded according to the result of the *post-hoc t*-test (*p < 0.05, **p < 0.01, and ***p < 0.001).

**Figure 6:**
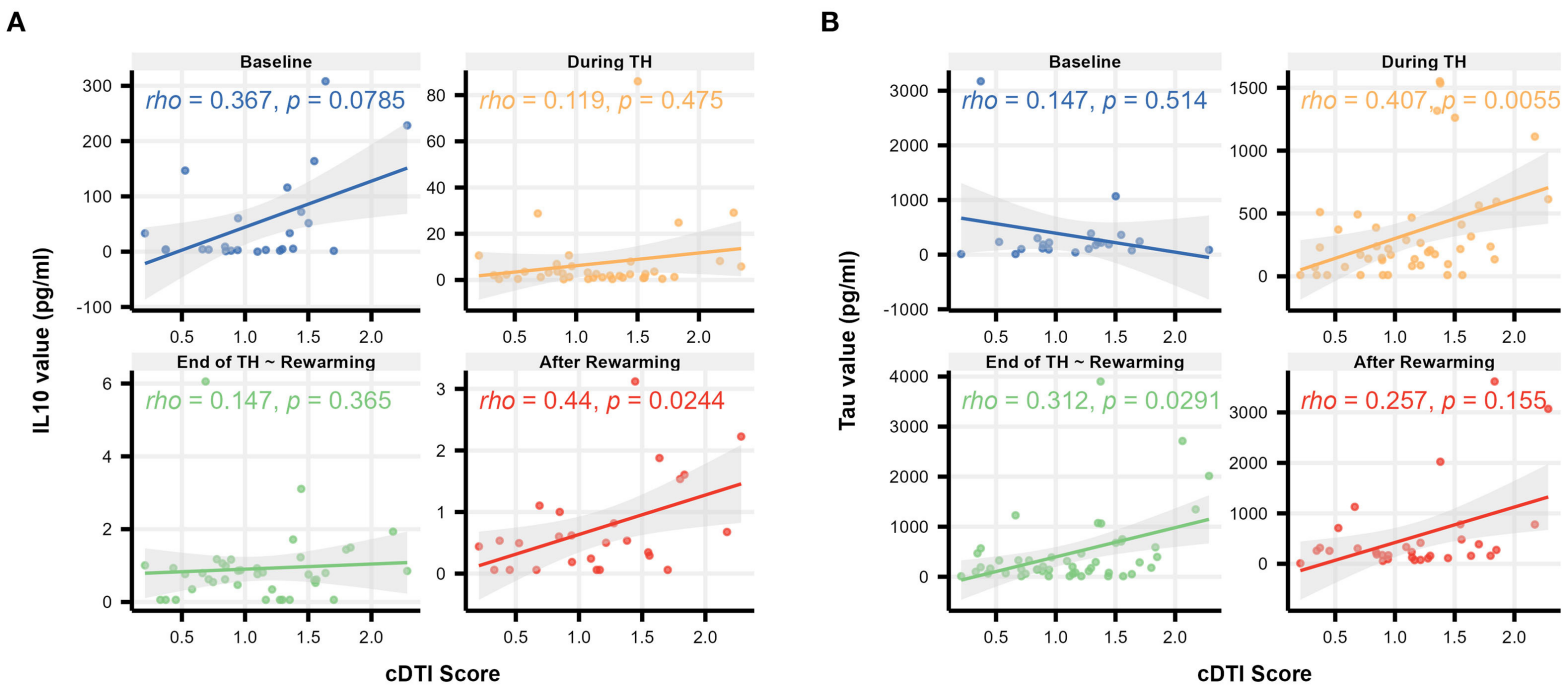
Scatterplots illustrating the relationship between the cDTI score and biomarker values [**(A)**: IL-10 and **(B)**: tau] over time (baseline, during TH, end of TH/rewarming, and after rewarming). Solid lines with gray areas indicate the regression lines with 95% confidence intervals, and Spearman's correlation coefficients/*p*-values are shown in the upper left corner of each graph.

Table 8 summarizes the result of the subsequent pairwise Welch's *t*-tests for each biomarker. These *post-hoc* tests aimed to identify at which time point the difference between the mild and severe groups was observed. Among the biomarkers that had significant differences between mild and severe groups (IL-10, tau), significant differences were found at baseline (*p* = 1.5 × 10^−13^) for IL-10 and at the end of TH/rewarming (*p* = 0.036) and after rewarming (*p* = 0.0015) for tau. These group differences are observed in Figure 5, illustrating the time course of IL-10 and tau concentrations by the groups. The Spearman's rank correlation test indicated a moderate correlation between the cDTI score and IL-10 concentration after rewarming (rho = 0.44, *p* = 0.024) and between the cDTI score and tau concentration during TH (rho = 0.41, *p* = 0.0056) and at the end of TH/rewarming (rho = 0.31, *p* = 0.029; Figure 6).

**Table 8:** Results of Welch's *t*-test for the difference in biomarker values between the mild and severe groups defined by the cDTI score for each time point.

**Biomarker^a^**	**Timepoint**	**Estimated difference (mild - severe)**	**SE**	**df**	**T-ratio**	***p*-value^b^**
BDNF	Baseline	−209	561	147	−0.37	0.71
	During TH	248	410	112	0.60	0.55
	End of TH/Rewarming	500	406	111	1.2	0.22
	After Rewarming	−103	468	133	−0.22	0.83
IL-6	Baseline	105	103	148	1.0	0.31
	During TH	−9.6	71	141	−0.14	0.89
	End of TH/Rewarming	−8.7	70	140	−0.13	0.90
	After Rewarming	−19	83	146	−0.23	0.82
VEGF	Baseline	−103	74	129	−1.4	0.17
	During TH	95	60	81	1.6	0.12
	**End of TH/Rewarming**	131	60	84	2.2	0.033*
	After Rewarming	122	65	102	1.9	0.064
GFAP	Baseline	0.088	0.93	131	0.095	0.92
	During TH	0.48	0.76	89	0.63	0.53
	End of TH/Rewarming	0.31	0.76	87	0.41	0.69
	After Rewarming	0.55	0.84	110	0.66	0.51
NRGN	Baseline	0.019	0.16	139	0.12	0.91
	During TH	−0.010	0.13	98	−0.077	0.94
	End of TH/Rewarming	0.12	0.13	96	0.89	0.38
	After Rewarming	0.13	0.15	120	0.86	0.39
**IL-10**	**Baseline**	−124	15	120	−8.3	**1.5** **×10**^−13^***
	During TH	−11	11	120	−1.0	0.30
	End of TH/Rewarming	0.013	11	120	0.0012	1.0
	After Rewarming	−0.49	13	120	−0.039	0.97
IL-8	Baseline	46	128	118	0.36	0.72
	During TH	59	96	92	0.61	0.54
	End of TH/Rewarming	58	98	94	0.59	0.56
	After Rewarming	−18	111	109	−0.16	0.88
**Tau**	Baseline	−50	310	140	−0.16	0.87
	During TH	−276	225	112	−1.2	0.22
	**End of TH/Rewarming**	−461	217	107	−2.1	0.036*
	**After Rewarming**	−808	250	126	−3.2	0.0015**

^a^Biomarkers with significant differences between the severe and mild groups are made bold.

^b^*p < 0.05, **p < 0.01, and ***p < 0.001.

### The effect of including different *b*-values

The results from the group of 56 patients, excluding the four patients who were scanned using a *b*-value of 800 s/mm^2^ to avoid the influence of the *b*-value on the DTI quantification, demonstrated a trend similar to that of the results that included all 60 patients. Although the number of anatomical structures selected by the LASSO regression model was slightly increased, all anatomical structures selected in the analysis of all 60 patients were included, and the magnitude of the regression coefficients for each anatomical structure tended to be similar (Supplementary Figure 2 and Supplementary Table 3). The significant correlations between the cDTI score and other severity scales (STO, NICHD-NRN, and Sarnat) and between the cDTI score and clinical variables were also unchanged (Supplementary Figures 3, 4 and Supplementary Tables 4, 5). In the mixed-model analysis of serum biomarkers and the cDTI scores, all significant effects (time point, severity, and interaction between both) remained except for the interaction effects in VEGF (Supplementary Table 6). Significant differences between severity groups were also replicated for IL-10 and tau concentrations.

## Discussion

### Clinical relevance of the cDTI score

We applied an atlas-based, whole-brain approach to capture the neuroradiological features of neonatal HIE that are associated with short-term neurological function. Our novel severity scale, the cDTI score, has the potential to resolve conventional challenges. It has been extensively reported that even cases with a severe clinical prognosis have subtle or no abnormalities on DWI or conventional MRI, leading to an increase in false negatives in the early diagnosis of HIE brain injury (Liauw et al., 2009; Rollins et al., 2014; Krishnan and Shroff, 2016; ElBeheiry et al., 2019). This conclusion is also supported by this study, in which more than 20% of the patients scored low (0, 1A, 1B) on the NICHD-NRN score, but were found to have severe short-term functional impairments (STO score of 3 or 4). On the contrary, the cDTI scores were strongly correlated with the STO scores, even in cases with the NICHD-NRN scores of 0, 1a, and 1b. However, the interpretation of the cDTI scores of 1–2 remained difficult. Nevertheless, our cDTI score may overcome one of the previous challenges of finding severe cases that were difficult to detect by visual qualitative MRI evaluation and may provide an accurate prediction of short-term clinical prognosis for those cases.

### Effect of variations in scanner and scan protocols

Although DTI is an essential imaging tool in terms of its ability to assess the brain quantitatively and is widely used in clinical research, it is necessary to consider the impact of different protocols on its quantitative data. For the acquisition of DTI parameters, we had matched voxel sizes (1 × 1 × 2 mm), but other parameters (scanner type, magnetic field strength, and *b*-value) could not be completely standardized; therefore, the results need to be interpreted carefully. Several studies have investigated the effect of these different protocols on FA/MD values. To assess the impact of using different scanners, Zhou et al. examined inter-manufacturer (GE vs. Siemens) and intra-manufacturer (Siemens Skyra vs. Siemens TrioTim) comparability and concluded there was a little impact between scanners on DTI metrics within the same manufacturer (Zhou et al., 2018), which we confirmed in this study. Regarding the magnetic field strength, although DTI theoretically is not affected by magnetic field strength, some papers report that it is affected (Huisman et al., 2006; Chung et al., 2013), while others conclude that it is not affected (Hunsche et al., 2001; Alexander et al., 2006), indicating the effect of magnetic field strength on quantitative DTI values and its mechanisms are not fully understood. Given this situation, we used a proportional odds model to examine the uniformity of the distribution of the STO scores among scanners and between magnetic field strength to check that the effect between protocols was not significant. The pairwise Spearman's correlation test on categorical clinical variables showed no significant correlation between the cDTI/STO scores and field strength or scanner preference, and the correlation coefficients were, at most, 0.14, which supports the small effect size of the protocol difference.

In terms of the difference in *b*-values, several studies have suggested that the signal-to-noise ratio decreases as *b*-value increases (Bisdas et al., 2008; Chung et al., 2013). Diffusivity measures are also known to be affected by *b*-value (Barrio-Arranz et al., 2015). Taking these findings into consideration, we performed the same series of analyses, with the exclusion of the four patients with an 800 s/mm^2^ *b*-value, and found that most of the results were similar to those before the exclusion, as shown in Section “The effect of including different b values” (Supplementary Figures 2–7 and Supplementary Tables 3–7). Although the impact of different *b*-values on the cDTI score was minor in this study, the influence of *b*-values on neurological prediction needs to be examined further.

### DTI measurements selected by LASSO regression model

Throughout the LASSO regression, we identified two types of relationships between the neurological functions of HIE and DTI measures: for structures such as the lateral frontotemporal structures and the cerebral peduncle, decreased MD values were related to the poor neurological functions of HIE, whereas for other structures including the limbic system, decreased FA and increased MD values were associated with the poor neurological functions of HIE. Although the pathogenetic factors responsible for the alterations in FA and MD after neonatal HIE remain unknown, these results may be explained by multiple mechanisms (Wu et al., 2014), namely, cytotoxic edema for the former group of structures, vasogenic edema for the latter group of structures, and the potential effects of Wallerian degeneration (Groenendaal et al., 2006), which can be observed in the subacute phase of HIE (Neil and Inder, 2006).

The primary pathogenesis of HIE can be broadly divided into ischemic and reperfusion phases. Cytotoxic edema occurs during the ischemic phase, whereas vasogenic edema results from the effects of free radical-induced vascular endothelial damage during the reperfusion phase (Distefano and Praticò, 2010). In our study, DTI was acquired during the subacute phase, in which the effects of both the ischemic and reperfusion phases could be observed. Gutierrez et al. demonstrated that, in the subacute phase of ischemia, blood reperfusion induces vasogenic edema, whereas the restriction of water mobility due to high blood viscosity also causes the exacerbation of cytotoxic edema (Gutierrez et al., 2010).

Cytotoxic edema is a redistribution of water from the extracellular to the intracellular space due to the disruption of the Na^+^/K^+^ ATPase pump and intracellular calcium concentration maintenance mechanisms, caused by decreased oxygen and glucose due to reduced cerebral blood flow (Allen and Brandon, 2011). This condition is known to cause decreased MD values due to cell swelling (Rai et al., 2008). On the contrary, in vasogenic edema, disruption of the blood–brain barrier (BBB) and increased vascular permeability leads to extravascular leakage of serum proteins, resulting in extracellular fluid retention (Utsunomiya, 2011). As a result, unlike cytotoxic edema, increased MD and decreased FA values are observed due to the expansion of the extracellular compartment (Keller et al., 1999; Moritani et al., 2000). In addition to these pathological mechanisms, our findings may also reflect the early impact of Wallerian degeneration on the corticospinal tracts. In the early stage of the degeneration (onset to 1–2 weeks), the axonal and myelin debris causes restriction of water diffusion, resulting in a significant decrease in FA or MD values of the corticospinal tracts (Yu et al., 2009; Qin et al., 2012). The posterior limb of the internal capsule and cerebral peduncle have been reported as preferential sites of the degeneration (Venkatasubramanian et al., 2013), which is consistent with the present results.

Among the structures identified in this study that may be involved in vasogenic edema, limbic-related structures, in particular, are consistent with the recently reported findings that they are susceptible to hypoxic–ischemic injury in neonates (Zheng et al., 2021; Parmentier et al., 2022). Long-term studies have identified survivors of even mild HIE, without evidence of cerebral palsy, to have deficits in functions served by the limbic system, including memory, emotional processing, and behavior (van Handel et al., 2010; Lee-Kelland et al., 2020), which is also reported in preclinical studies (Burnsed et al., 2015; Diaz et al., 2017; Chavez-Valdez et al., 2018). Another study of 10-year-olds with a history of neonatal HIE found that smaller mammillary body and hippocampal volumes were associated with lower total IQ, performance IQ, processing speed, and episodic memory (Annink et al., 2021). Our results support that the severity of impairment of the limbic network by HIE may be related to the severity of neurological sequelae. Preclinical animal studies also support regionally specific temporal patterns of cell death and neurodegeneration (Northington et al., 2001a,b, 2022; Chavez-Valdez et al., 2021a). Thus, the results also suggested that the progression of cytotoxic and vasogenic edema and Wallerian degeneration occurs heterogeneously in different brain regions and that the pattern of progression and the severity may be associated with neurological functions.

### Relationship between cDTI score and time course of serum biomarker

We also examined the potential biological mechanism supporting the cDTI score by comparing the time course of serum biomarker values between the cDTI score-defined severe and mild disease groups. The results of the mixed-model analysis showed that not only higher cDTI scores were associated with higher IL-10 and tau values, but also, more precisely, at which time points these relationships were observed: at baseline for IL-10 and at the end of TH/rewarming and after rewarming for tau. Moreover, tau was correlated with the cDTI score during TH and at the end of TH/rewarming, further supporting its association.

Our group has previously reported in a larger cohort, which included those patients studied here, that indicators of worse HIE severity, including the Sarnat scores correlated with higher IL-10 within the first 24 h of life, and tau at and after TH (Chavez-Valdez et al., 2021b). Increased levels of IL-10 (Orrock et al., 2016) and tau proteins (Dietrick et al., 2020) are closely linked to the worse prognosis of HIE. Previous animal (Li et al., 2014; Bai et al., 2021) and human (Youn et al., 2013) studies have identified a broad anti-inflammatory role for IL-10, such as the prevention of pro-inflammatory cytokine synthesis and the excessive phosphorylation of tau protein that leads to microtubule collapse and deposition in neurons, resulting in neurodegeneration (Wu et al., 2017c). Interpreting the present results in light of these findings, the cDTI score may be a composite measure that reflects the degree of both inflammatory responses in the early stages and accumulated neuronal damage in the later stages in HIE patients with TH.

### Relationship between cDTI and STO scores

In a previous study by our group, neonates suffering from HIE who end up developing qualitative MRI evidence of brain injury despite TH took an average of 17 ± 9 days to reach full oral feeds, with >60% needing G-tube placement prior to discharge, and >10% dying, which contrasted with those HIE neonates with negative MRI, who took only 9 ± 5 days to reach full feeds and had no need for G-tube or mortality (Ennen et al., 2011). Others have also found similar feeding difficulties in this population (Krüger et al., 2019) and associations with brain injury in MRI and alterations in EEG (Badran et al., 2020; Takle et al., 2021). Thus, time to reach full oral feeds, the need for G-tube, and mortality after TH are appropriate functional assessments of the severity of short-term neurological functions and were used to build the STO score as described in Section “Short-term oral-feeding (STO) score.” Here, we show that while the Sarnat or NICHD-NRN scores alone have a weak correlation with the STO scores, the cDTI score has a strong correlation, which was not related to sex, field strength, MRI scanner, or other clinical variables. Furthermore, unlike low cDTI scores, low NICHD-NRN scores do not necessarily relate to better neurological functions based on the STO scores. Therefore, the cDTI scores are superior in identifying poor neurological functions in HIE neonates without major abnormalities on qualitative MRI reading, suggesting a higher sensitivity in detecting mild neuronal damage.

### Limitations

Our study has several limitations. First, the scanner and scan protocols varied among patients. We are fully aware of the impact of differences in scanners, magnetic field strength, and *b*-values on DTI measurements. Although our results indicated that such technological variations were negligible in obtaining cDTI scores, the potential impact of the technological variations on the predictive model and cDTI scores cannot be completely excluded. Nevertheless, the results suggest that the cDTI score is a robust measure against technological variability, and this robustness is crucial in clinical applications across institutions. Second, our cohort was comprised predominantly of neonates with mild-to-moderate rather than moderate-to-severe HIE injury by MRI, with relatively small contributions from basal ganglia and thalamic structures known to be involved in moderate-to-severe HIE before TH became standard of care for this subgroup of patients. Whether the cDTI scores from our model can predict the neurological function in more severely injured patients remains to be explored. Third, our analysis did not include standard neurological assessments such as the Hammersmith Neonatal Neurological Assessment, the NICU Network Neurobehavioral Scale, or the General Movements Assessment. Although an association between oral feeding difficulties and poor neurological prognosis has been reported, a direct comparison of the STO scores with gold standard assessments has not been made. Future studies are needed to test whether the STO scores accurately reflect short- and long-term neurological complications. Particularly, a longer follow-up is needed to assess whether the cDTI score is predictive of future neurological function. Fourth, the current single atlas analysis required manual correction to quantify DTI-derived scalar indices when errors in parcellation occur. This need for manual correction may limit its use in clinical practice. We are currently developing an atlas-based image parcellation tool based on the multi-atlas label fusion (MALF) algorithm that uses multiple atlases as teaching files to accurately parcellate neonatal brains (Tang et al., 2014; Otsuka et al., 2019). MALF algorithm, once validated, is expected to improve the accuracy of image parcellation and enable fully automated DTI quantification suitable for clinical applications. Fifth, a substantial number of patients in the original study cohort did not undergo brain MRI scans during their NICU admission. Because our study included only neonates who had MRI scans, there may be a patient selection bias based on indications or contraindications for brain MRI scans. The cohort in this study had relatively mild Sarnat scores, which may have excluded HIE neonates with unstable clinical conditions that precluded them from undergoing MRI scans. Finally, although our cohort size was larger than previous studies using DTI in neonates, the number of participants was still small, considering the number of regions analyzed in a whole-brain approach. Larger cohorts may allow for more stable modeling with less prediction error and may identify more areas associated with disease severity.

### Summary

Using an unbiased composite quantitative imaging measure across whole-brain structures, we developed the cDTI score, a novel severity measurement correlating with short-term neurological status in HIE patients who undergo TH. Limbic and frontotemporal regions and corticospinal projection fibers were identified as a lesion associated with short-term neurological functions. The relationship between the cDTI score and serum biomarkers suggested that the cDTI score reflects the inflammatory response prior to TH and the neuronal damage observed after TH. Larger studies that include more patients with moderate-to-severe HIE are needed to validate the cDTI score and assess how it can be implemented in clinical practice.

## Data availability statement

The original contributions presented in the study are included in the article/Supplementary material, further inquiries can be directed to the corresponding author.

## Ethics statement

This study was reviewed and approved by Johns Hopkins School of Medicine IRB. Written informed consent to participate in this study was provided by the participants' legal guardian/next of kin.

## Author contributions

EC, RC-V, AE, FN, and KOi were involved in conceptualization and design of the study. KOn, EC, RC-V, and KOi were involved in methodology and drafted the significant portions of manuscript, tables, and figures. RC-V, AM, BS, AT, CP, DV, EG, AE, CS, FN, and KOi were involved in supervision and oversight. KOn, EC, JC, and KOi were involved in formal data analysis. RC-V, AM, BS, HS, SM, AT, CP, DV, EG, AE, and FN were involved in resources. All authors were involved in data acquisition, reviewing, and editing of the final manuscript.

## Funding

This work was supported by the National Institutes of Health R01HD065955 (KOi), R01NS126549 (KOi), RO1HD086058 (AE and FN), RO1HD070996 (FN), R21AG061643 (FN), K08NS096115 (RC-V), and K08NS096115-03S1 (RC-V); the JHU-SOM Clinician Scientist Award (RC-V); and the Thomas Wilson Foundation (RC-V).

## Conflict of interest

KOi is a consultant for “AnatomyWorks” and “Corporate-M.” This arrangement is being managed by the Johns Hopkins University in accordance with its conflict-of-interest policies. The remaining authors declare that the research was conducted in the absence of any commercial or financial relationships that could be construed as a potential conflict of interest.

## Publisher's note

All claims expressed in this article are solely those of the authors and do not necessarily represent those of their affiliated organizations, or those of the publisher, the editors and the reviewers. Any product that may be evaluated in this article, or claim that may be made by its manufacturer, is not guaranteed or endorsed by the publisher.
