# Supplementary material for: Development of a composite diffusion tensor imaging score correlating with short-term neurological status in neonatal hypoxic–ischemic encephalopathy
Source: Front Neurosci. 2022 Aug 2;16:931360. doi: 10.3389/fnins.2022.931360 (PMC9379310; doi:10.3389/fnins.2022.931360)
Supplement: Supplementary file 1 [file Data_Sheet_1.docx]

Supplementary Material

## Supplementary Figures

**Supplementary Figure 1.** Scatterplots showing the relationship between the cDTI score and the selected 17 factors through the LASSO regression. Solid black lines with gray areas represent the regression lines with 95% confidence intervals, and Spearman’s correlation coefficients/p-values are shown in the upper left corner of each graph. Factors are ordered from largest to smallest LASSO regression coefficient.

**
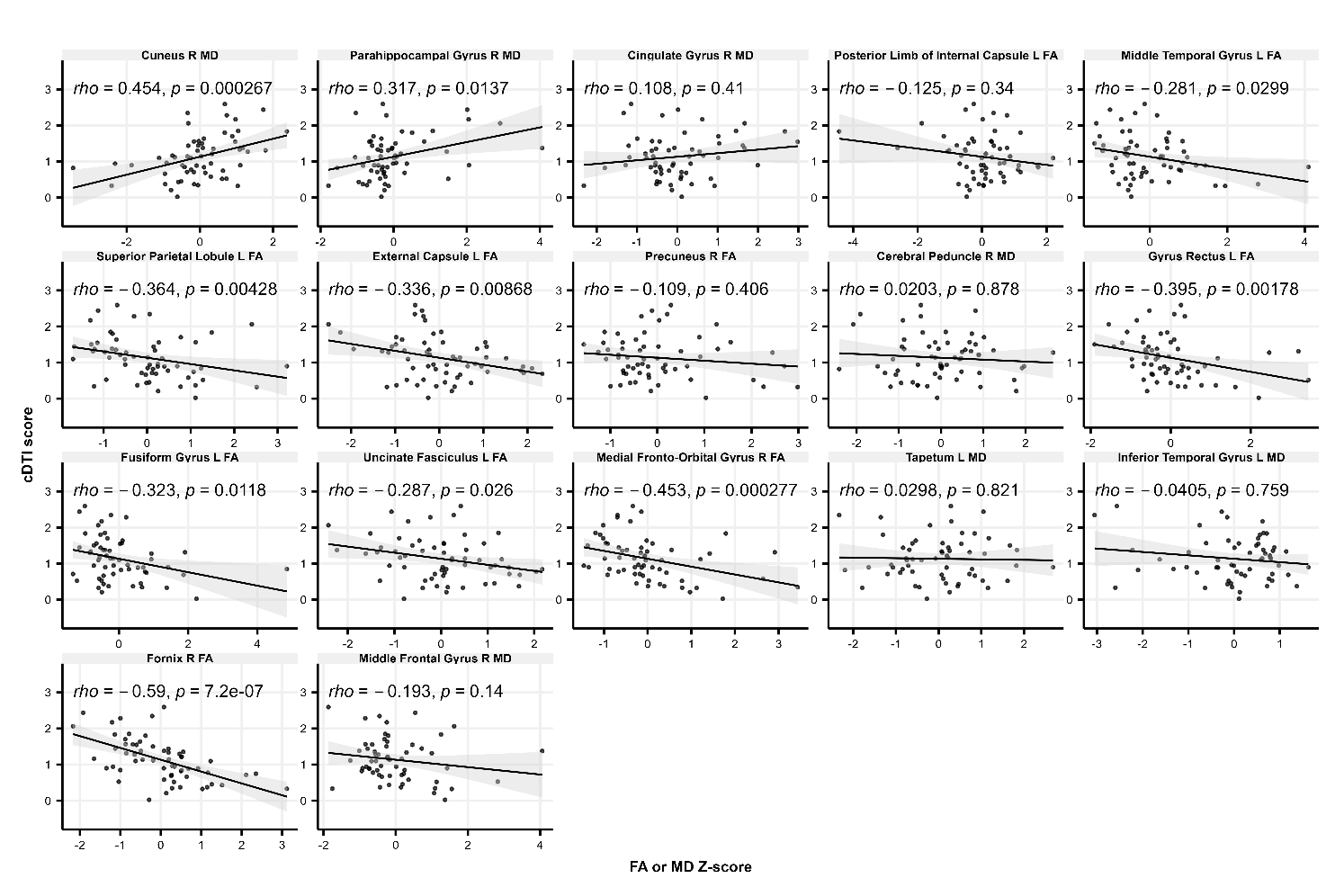
**

**Supplementary Figure 2.** Representative parcellation maps superimposed on FA maps (after b-value adjustment). The locations and laterality (L/R) of the selected 20 structures in the cDTI score calculation are annotated. (A-D) Axial images at the level of the corticospinal tract, the uncinate fasciculus (A), the cerebral peduncle (B), the basal ganglia (C), and the superior parietal lobule (D). (E-F) Sagittal images at the level of the right fornix, the right cuneus (E), and the left corpus callosum (F).


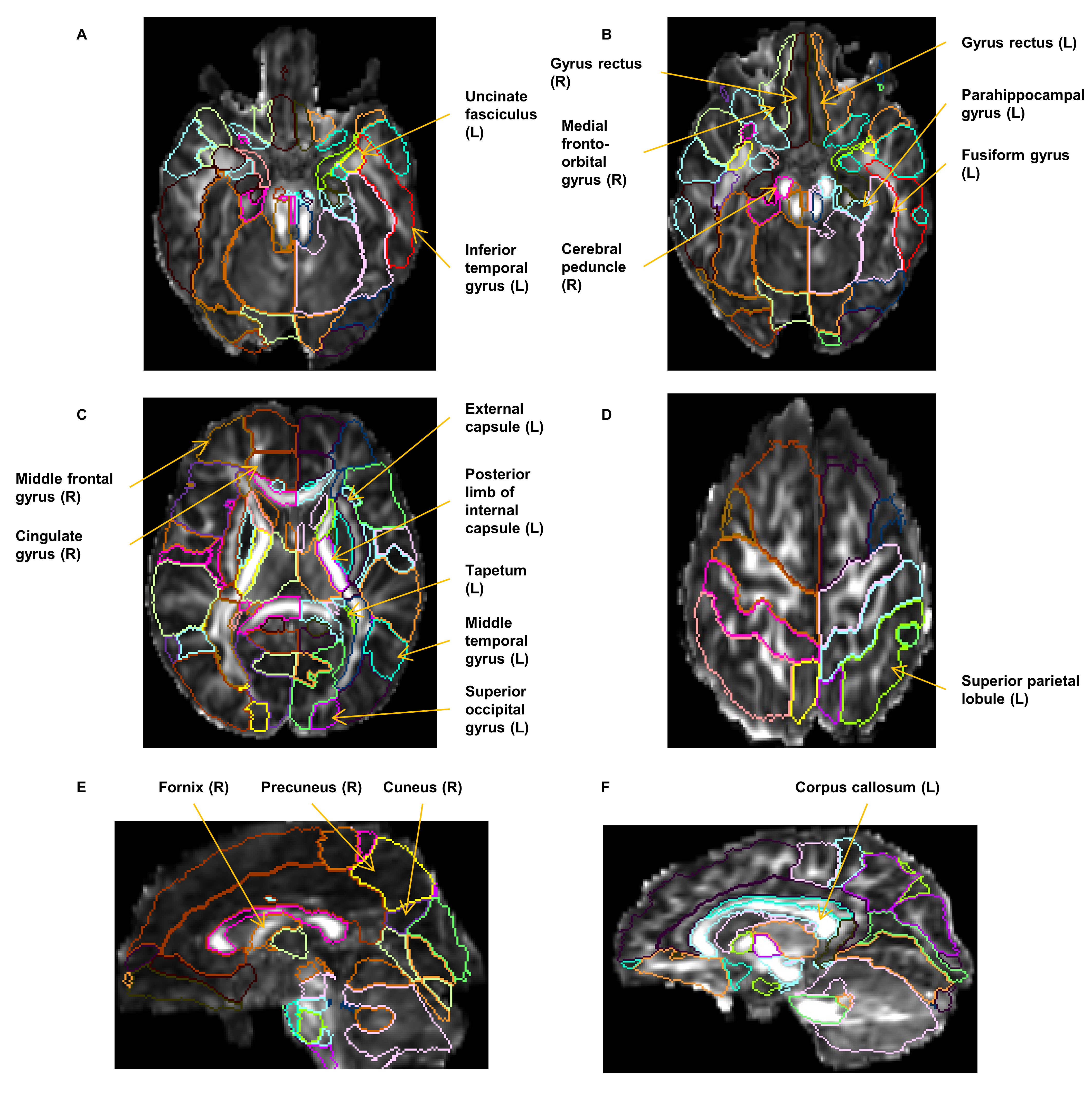


**Supplementary Figure 3.** Scatterplots showing the relationship among severity scales (after b-value adjustment). (A) Comparison between the cDTI score and the STO score, (B) the NICHD-NRN score and the STO score, and (C) the NICHD-NRN score and the cDTI score. Solid black lines with gray areas represent the regression lines with 95% confidence intervals, and Spearman’s correlation coefficients/p-values are shown in the upper left corner of each graph. For (B), the data are jittered to show the sample size.


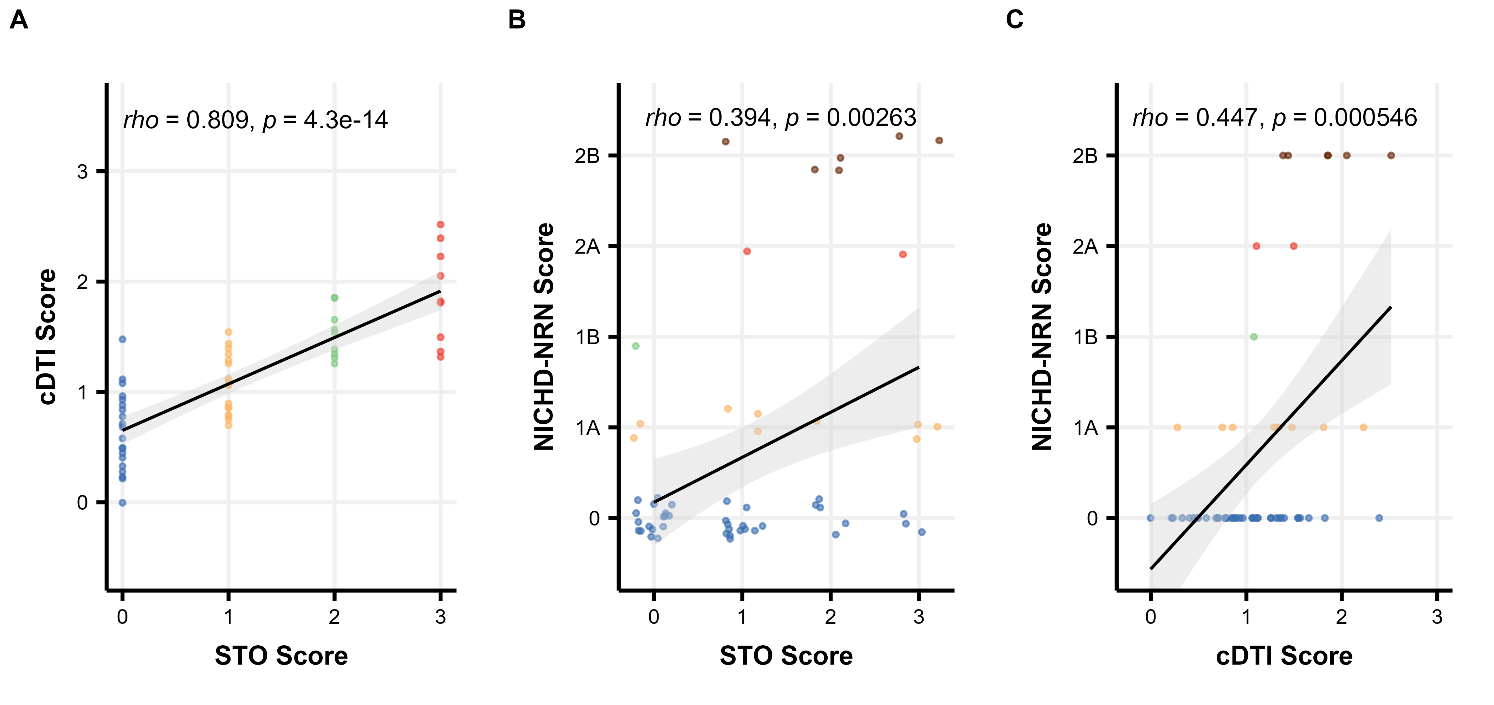


**Supplementary Figure 4.** (A) Spearman's correlation coefficient matrix between cDTI score and categorical clinical variables (STO, Sarnat, NICHD-NRN, Sex, Field strength, Scanner preference). (B) Pearson's correlation coefficient matrix between cDTI scores and numerical clinical variables (postmenstrual age, menstrual age, gestational age, and weight). Color-coded numbers in the upper right half of the matrix indicate correlation coefficients (*p-value < 0.05, **p-value < 0.01, ***p-value < 0.001, blue: positive coefficient, red: negative coefficient). The color-coded ellipses in the lower left half of the matrix indicate the strength of correlation between variables, with blue indicating negative correlation and red indicating positive correlation. The shape of the ellipses indicates the strength of the correlation (ellipses are sharp when the correlation is strong and round when it is weak), positive slope indicates positive correlation and negative slope indicates negative correlation.


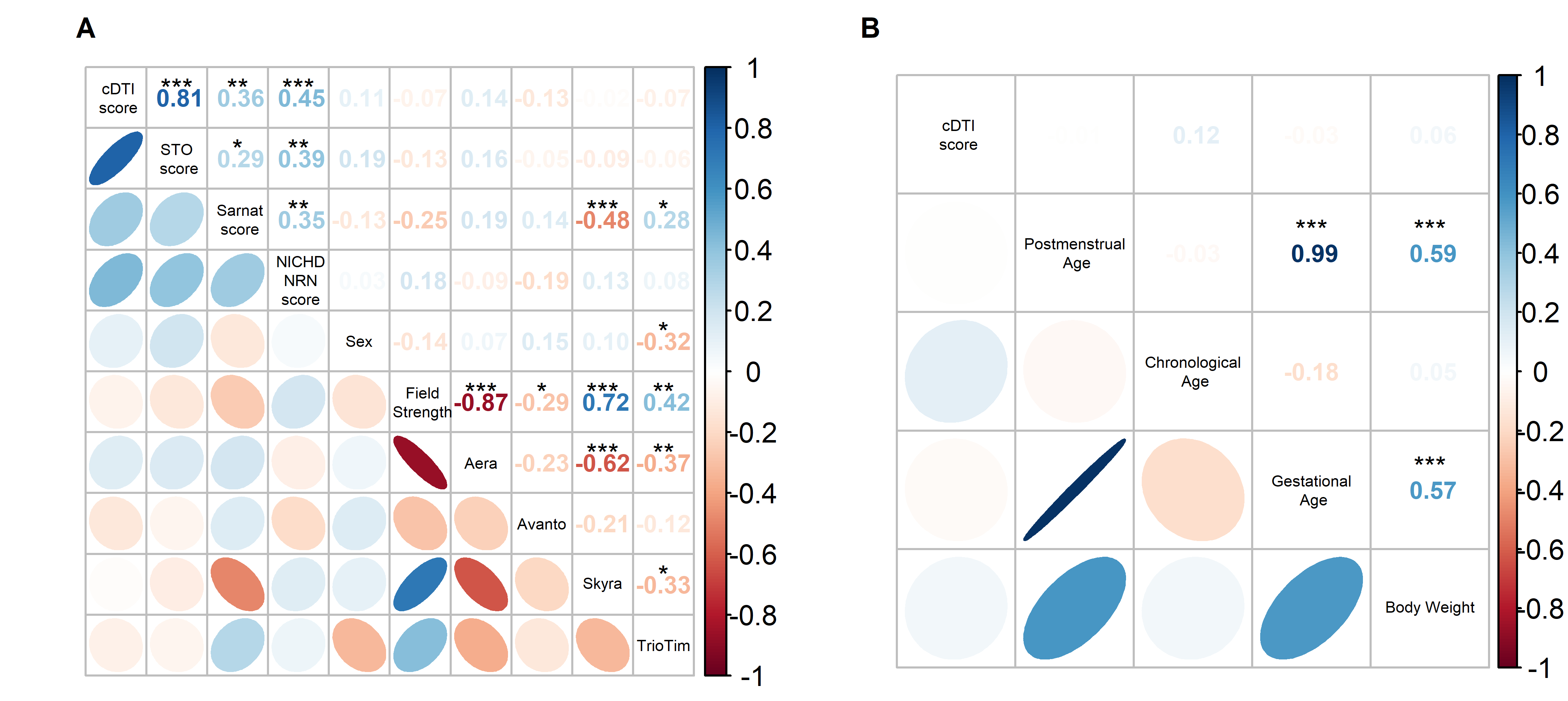


**Supplementary Figure 5.** Time courses of biomarker values (A: IL-10, B: Tau) by severity group (after b-value adjustment). Raw biomarker values are shown as scatterplots and time courses are indicated as error bars. The error bars on each timepoint (baseline, during TH, end of TH/rewarming, and after rewarming) were calculated based on the results of mixed model analysis. Significance stars are embedded according to the results of the post hoc t-test (*p-value < 0.05, ***p-value < 0.001).


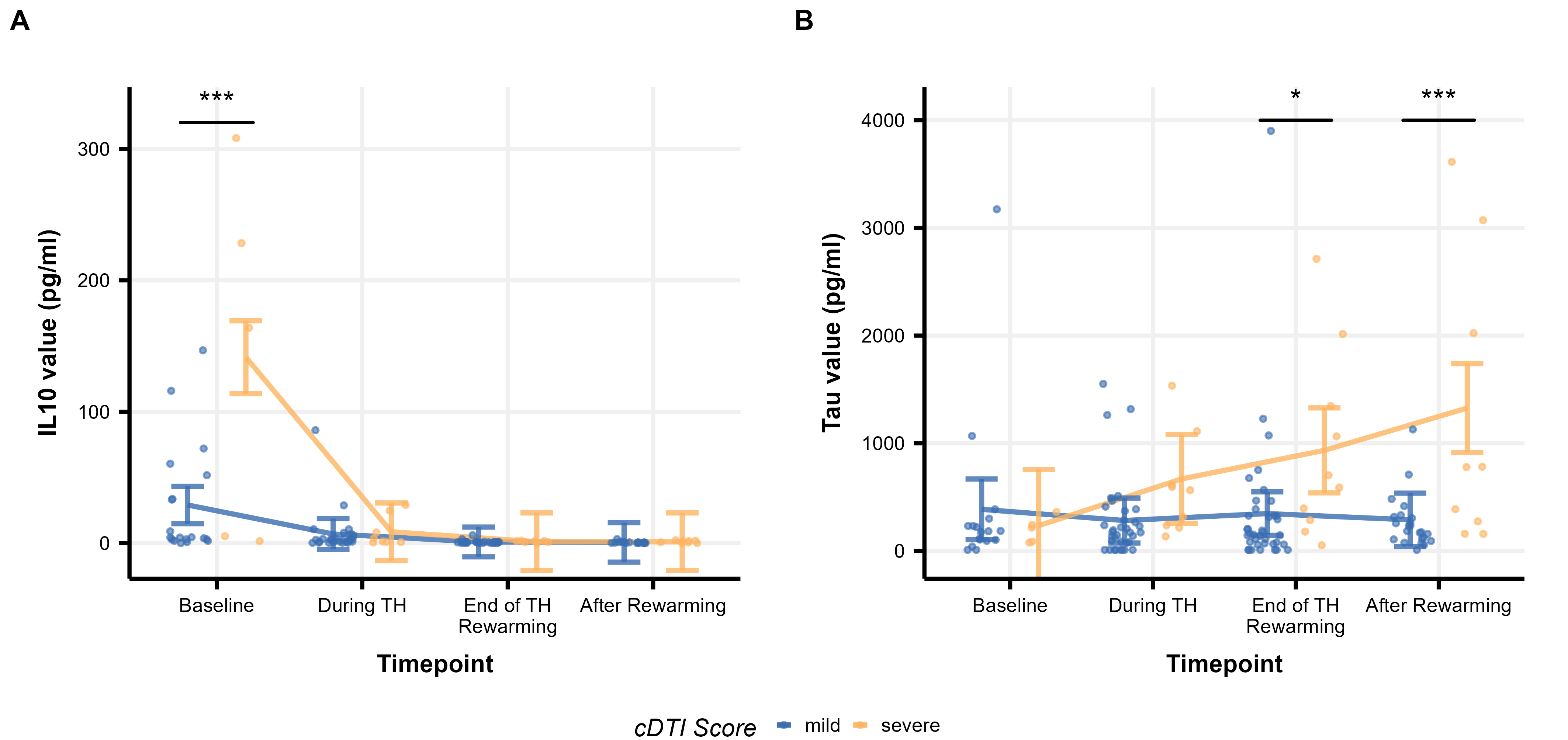


**Supplementary Figure 6.** Scatter plots illustrating the relationship between the cDTI score and biomarker values (A: IL-10, B: Tau) over time (baseline, during TH, end of TH/rewarming, and after rewarming) (after b-value adjustment). Solid lines with gray areas indicate the regression lines with 95% confidence intervals, and Spearman’s correlation coefficients/p-values are shown in the upper left corner of each graph.


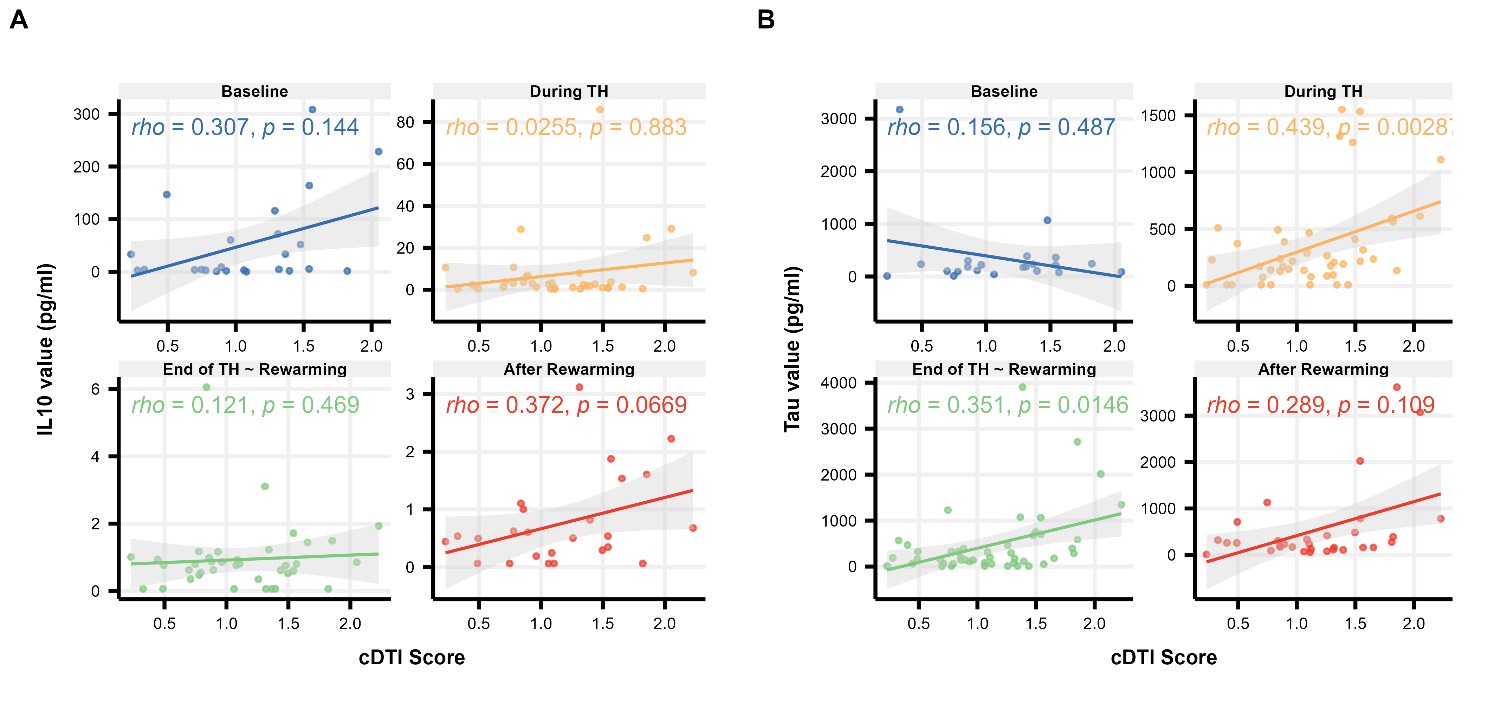


**Supplementary Figure 7.** Scatterplots showing the relationship between the cDTI score and the selected 20 factors through the LASSO regression (after b-value adjustment). Solid black lines with gray areas represent the regression lines with 95% confidence intervals, and Spearman’s correlation coefficients/p-values are shown in the upper left corner of each graph. Factors are ordered from the largest to smallest LASSO regression coefficient.
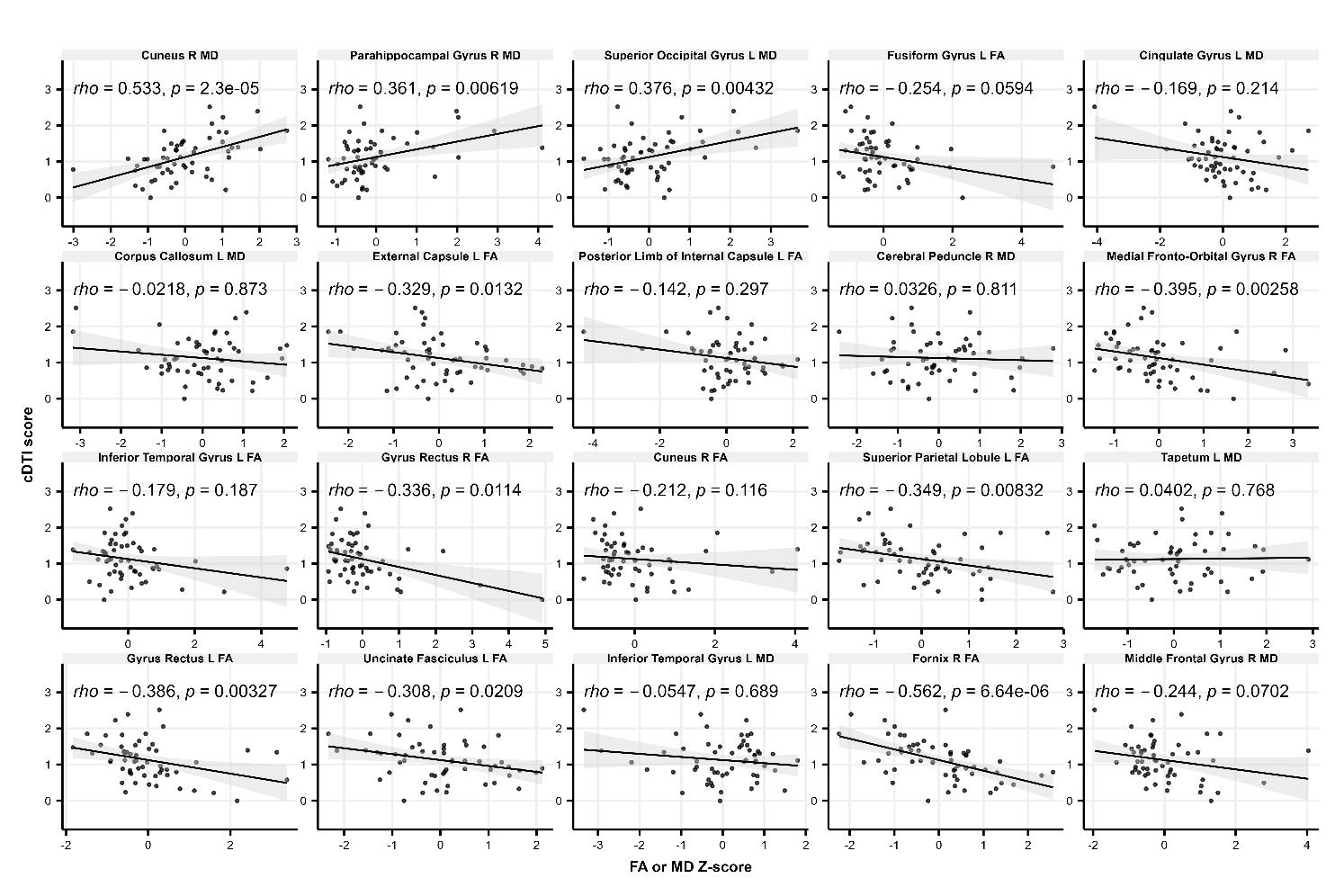


## Supplementary Tables

**Supplementary Table 1.** Results of the proportional odds model analysis for evaluation of the STO score distribution between two levels of field strength (1.5T/3T).

Analysis of Deviance Table (Type II tests)

| **Variable** | **χ ^2^** | **Df** | **p-value** |
| --- | --- | --- | --- |
| Field Strength | 0.76 | 1 | 0.38 |

Brant Test Table for the proportional odds model

| **Variable tested for** | **χ ^2^** | **df** | **p-value^1^** |
| --- | --- | --- | --- |
| Field Strength 1.5T | 0.15 | 2 | 0.93 |
| Field Strength 3T | 0.15 | 2 | 0.93 |

*^1^If there are no significant p-values, the model satisfies the proportional odds assumption.*

**Supplementary Table 2.** The list of 100 structures eligible for the cDTI score calculation model.

|  | Structure | L/R |  | Structure | L/R |
| --- | --- | --- | --- | --- | --- |
| 1 | Corpus callosum | L | **51** | Pons | L |
| 2 | Corpus callosum | R | **52** | Pons | R |
| 3 | Anterior limb of internal capsule | L | **53** | Medial lemniscus | L |
| 4 | Anterior limb of internal capsule | R | **54** | Medial lemniscus | R |
| 5 | Posterior limb of internal capsule | L | **55** | Superior frontal gyrus | L |
| 6 | Posterior limb of internal capsule | R | **56** | Superior frontal gyrus | R |
| 7 | Retrolenticular part of internal capsule | L | **57** | Middle frontal gyrus | L |
| 8 | Retrolenticular part of internal capsule | R | **58** | Middle frontal gyrus | R |
| 9 | Superior corona radiata | L | **59** | Medial fronto-orbital gyrus | L |
| 10 | Superior corona radiata | R | **60** | Medial fronto-orbital gyrus | R |
| 11 | Posterior corona radiata | L | **61** | Lateral fronto-orbital gyrus | L |
| 12 | Posterior corona radiata | R | **62** | Lateral fronto-orbital gyrus | R |
| 13 | Cingulum cingular part | L | **63** | Gyrus rectus | L |
| 14 | Cingulum cingular part | R | **64** | Gyrus rectus | R |
| 15 | Cingulum hippocampal part | L | **65** | Precentral gyrus | L |
| 16 | Cingulum hippocampal part | R | **66** | Precentral gyrus | R |
| 17 | Fornix | L | **67** | Postcentral gyrus | L |
| 18 | Fornix | R | **68** | Postcentral gyrus | R |
| 19 | Stria terminalis | L | **69** | Superior parietal gyrus | L |
| 20 | Stria terminalis | R | **70** | Superior parietal gyrus | R |
| 21 | Tapetum | L | **71** | Precuneus | L |
| 22 | Tapetum | R | **72** | Precuneus | R |
| 23 | External capsule | L | **73** | Cingular gyrus | L |
| 24 | External capsule | R | **74** | Cingular gyrus | R |
| 25 | Posterior thalamic radiation | L | **75** | Supramarginal gyrus | L |
| 26 | Posterior thalamic radiation | R | **76** | Supramarginal gyrus | R |
| 27 | Sagittal stratum | L | **77** | Angular gyrus | L |
| 28 | Sagittal stratum | R | **78** | Angular gyrus | R |
| 29 | Thalamus | L | **79** | Superior temporal gyrus | L |
| 30 | Thalamus | R | **80** | Superior temporal gyrus | R |
| 31 | Putamen | L | **81** | Middle temporal gyrus | L |
| 32 | Putamen | R | **82** | Middle temporal gyrus | R |
| 33 | Caudate nucleus | L | **83** | Inferior temporal gyrus | L |
| 34 | Caudate nucleus | R | **84** | Inferior temporal gyrus | R |
| 35 | Cerebral peduncle | L | **85** | Fusiform gyrus | L |
| 36 | Cerebral peduncle | R | **86** | Fusiform gyrus | R |
| 37 | Inferior fronto-occipital fasciculus | L | **87** | Parahippocampal gyrus | L |
| 38 | Inferior fronto-occipital fasciculus | R | **88** | Parahippocampal gyrus | R |
| 39 | Corticospinal tract | L | **89** | Superior occipital gyrus | L |
| 40 | Corticospinal tract | R | **90** | Superior occipital gyrus | R |
| 41 | Superior cerebellar peduncle | L | **91** | Middle occipital gyrus | L |
| 42 | Superior cerebellar peduncle | R | **92** | Middle occipital gyrus | R |
| 43 | Middle cerebellar peduncle | L | **93** | Cuneus | L |
| 44 | Middle cerebellar peduncle | R | **94** | Cuneus | R |
| 45 | Inferior cerebellar peduncle | L | **95** | Lingual gyrus | L |
| 46 | Inferior cerebellar peduncle | R | **96** | Lingual gyrus | R |
| 47 | Uncinate fasciculus | L | **97** | Hippocampus | L |
| 48 | Uncinate fasciculus | R | **98** | Hippocampus | R |
| 49 | Midbrain | L | **99** | Cerebellar hemisphere | L |
| 50 | Midbrain | R | **100** | Cerebellar hemisphere | R |

**Supplementary Table 3.** Regression coefficients of the 20 factors selected through the LASSO regression analysis (after b-value adjustment).

| Positive Regression Coefficients | | | |
| --- | --- | --- | --- |
| Regression Coefficient | **DTI** | **Anatomical Structure** | Side |
| 0.30 | MD | Cuneus* | Right |
| 0.082 | MD | Parahippocampal gyrus*† | Right |
| 0.060 | MD | Superior occipital gyrus* | Left |
| Negative Regression Coefficients | | | |
| Regression Coefficient | **DTI** | **Anatomical Structure** | Side |
| -0.35 | MD | Middle frontal gyrus | Right |
| -0.18 | FA | Fornix*† | Right |
| -0.13 | MD | Inferior temporal gyrus | Left |
| -0.10 | FA | Uncinate fasciculus*† | Left |
| -0.092 | FA | Gyrus rectus* | Left |
| -0.069 | MD | Tapetum | Left |
| -0.061 | FA | Superior parietal lobule* | Left |
| -0.048 | FA | Cuneus | Right |
| -0.047 | FA | Gyrus rectus* | Right |
| -0.041 | FA | Inferior temporal gyrus | Left |
| -0.033 | FA | Medial fronto-orbital gyrus*† | Right |
| -0.032 | MD | Cerebral peduncle | Right |
| -0.030 | FA | Posterior limb of internal capsule | Left |
| -0.016 | FA | External capsule* | Left |
| -0.016 | MD | Corpus callosum | Left |
| -0.014 | MD | Cingulate gyrus† | Left |
| -0.011 | FA | Fusiform gyrus | Left |

**Significant correlation with cDTI score (Spearman’s correlation, p < 0.05).*

†*Limbic fibers and related structures.*

**Supplementary Table 4.** Spearman’s correlation coefficients and p-values between the cDTI score and the categorical clinical variables (after b-value adjustment).

| **Statistic** | **STO**  **score** | **Sarnat score** | **NICHD**  **NRN**  **score** | **Sex** | **Field Strength** | **Aera** | **Avanto** | **Skyra** | **Trio Tim** |
| --- | --- | --- | --- | --- | --- | --- | --- | --- | --- |
| rho | **0.81** | **0.36** | **0.45** | 0.11 | -0.070 | 0.14 | -0.13 | -0.018 | -0.071 |
| p-value | **4.3×10^-14^***** | **0.0067**** | **5.5×10^-4^***** | 0.43 | 0.61 | 0.31 | 0.35 | 0.89 | 0.61 |

*^1^**p-value < 0.01, ***p-value < 0.001*

**Supplementary Table 5.** Pearson correlation coefficients and p-values between the cDTI score and the continuous clinical variables (after b-value adjustment).

| **Statistic** | **Postmenstrual age at MRI scan (weeks)** | **Chronological age at MRI scan (days)** | **Gestational age at birth (weeks)** | **Body weight at birth (grams)** |
| --- | --- | --- | --- | --- |
| r | -0.0094 | 0.12 | -0.026 | 0.058 |
| p-value | 0.95 | 0.40 | 0.85 | 0.67 |

**Supplementary Table 6.** Results of mixed model analysis for each biomarker (after b-value adjustment).

| **Biomarker^1^** | **Source^2^** | **df** | **df (residual)** | **F-value** | **p-value^3^** |
| --- | --- | --- | --- | --- | --- |
| BDNF | **Timepoint** | 3 | 102 | 4.1 | **0.0089**** |
|  | Severity | 1 | 46 | 0.55 | 0.46 |
|  | Timepoint * Severity | 3 | 100 | 1.1 | 0.36 |
| IL-6 | **Timepoint** | 3 | 108 | 3.0 | **0.033*** |
|  | Severity | 1 | 42 | 0.021 | 0.89 |
|  | Timepoint * Severity | 3 | 105 | 0.51 | 0.67 |
| VEGF | **Timepoint** | 3 | 97 | 5.7 | **0.0013**** |
|  | Severity | 1 | 47 | 1.3 | 0.25 |
|  | Timepoint * Severity | 3 | 96 | 2.3 | 0.085 |
| GFAP | **Timepoint** | 3 | 100 | 3.7 | **0.014*** |
|  | Severity | 1 | 47 | 0.54 | 0.47 |
|  | Timepoint * Severity | 3 | 99 | 1.0 | 0.39 |
| NRGN | Timepoint | 3 | 102 | 1.3 | 0.29 |
|  | Severity | 1 | 46 | 0.072 | 0.79 |
|  | Timepoint * Severity | 3 | 100 | 0.57 | 0.64 |
| **IL-10** | **Timepoint** | 3 | 90 | 17 | **1.1×10^-08^***** |
|  | **Severity** | 1 | 30 | 8.5 | **0.0066**** |
|  | **Timepoint * Severity** | 3 | 86 | 14 | **2.4×10^-07^***** |
| IL-8 | Timepoint | 3 | 83 | 1.8 | 0.15 |
|  | Severity | 1 | 35 | 0.046 | 0.83 |
|  | Timepoint * Severity | 3 | 80 | 0.18 | 0.91 |
| **TAU** | Timepoint | 3 | 99 | 1.2 | 0.33 |
|  | **Severity** | 1 | 44 | 9.7 | **0.0033**** |
|  | **Timepoint * Severity** | 3 | 97 | 4.6 | **0.0046**** |

*^1^Biomarkers with significant differences between the severe and mild groups are made bold.*

*^2^The Severity here is a binary variable of severity grouping (mild/severe) based on cDTI score. Timepoint*Severity cells shows interactions of timepoint and severity variables.*

*^3^*p-value < 0.05, **p-value < 0.01, ***p-value < 0.001*

**Supplementary Table 7.** Results of Welch's t-test for the difference in biomarker values between the mild and severe groups defined by the cDTI score for each time point (after b-value adjustment).

| **Biomarker^1^** | **Timepoint** | **Estimated**  **difference**  **(mild - severe)** | **SE** | **df** | **T-ratio** | **p-value^2^** |
| --- | --- | --- | --- | --- | --- | --- |
| BDNF | Baseline | -591 | 572 | 139 | -1.0 | 0.30 |
|  | During TH | -415 | 458 | 110 | -0.91 | 0.37 |
|  | End of TH/Rewarming | -394 | 439 | 104 | -0.90 | 0.37 |
|  | After Rewarming | 333 | 482 | 119 | 0.69 | 0.49 |
| IL-6 | Baseline | 104 | 105 | 143 | 0.99 | 0.33 |
|  | During TH | -42 | 80 | 136 | -0.53 | 0.60 |
|  | End of TH/Rewarming | -15 | 76 | 134 | -0.20 | 0.85 |
|  | After Rewarming | -25 | 85 | 139 | -0.30 | 0.77 |
| VEGF | Baseline | -67 | 80 | 115 | -0.84 | 0.41 |
|  | During TH | 96 | 68 | 80 | 1.4 | 0.16 |
|  | End of TH/Rewarming | 70 | 68 | 79 | 1.0 | 0.31 |
|  | After Rewarming | 108 | 71 | 88 | 1.5 | 0.13 |
| GFAP | Baseline | 0.45 | 0.96 | 114 | 0.47 | 0.64 |
|  | During TH | -0.38 | 0.86 | 86 | -0.44 | 0.66 |
|  | End of TH/Rewarming | -0.74 | 0.83 | 80 | -0.90 | 0.37 |
|  | After Rewarming | -1.0 | 0.88 | 93 | -1.2 | 0.24 |
| NRGN | Baseline | -0.015 | 0.17 | 125 | -0.086 | 0.93 |
|  | During TH | -0.066 | 0.15 | 96 | -0.44 | 0.66 |
|  | End of TH/Rewarming | 0.087 | 0.14 | 89 | 0.61 | 0.54 |
|  | After Rewarming | 0.096 | 0.15 | 103 | 0.62 | 0.53 |
| **IL-10** | **Baseline** | -112 | 16 | 115 | -7.0 | **1.6×10^-10^***** |
|  | During TH | -1.7 | 13 | 115 | -0.13 | 0.90 |
|  | End of TH/Rewarming | -0.16 | 13 | 115 | -0.013 | 0.99 |
|  | After Rewarming | -0.50 | 14 | 115 | -0.037 | 0.97 |
| IL-8 | Baseline | -31 | 132 | 108 | -0.23 | 0.82 |
|  | During TH | 62 | 111 | 87 | 0.56 | 0.58 |
|  | End of TH/Rewarming | 25 | 110 | 86 | 0.23 | 0.82 |
|  | After Rewarming | -8.8 | 117 | 94 | -0.076 | 0.94 |
| **TAU** | Baseline | 151 | 304 | 136 | 0.50 | 0.62 |
|  | During TH | -386 | 236 | 110 | -1.6 | 0.11 |
|  | **End of TH/Rewarming** | -587 | 226 | 105 | -2.6 | **0.011*** |
|  | **After Rewarming** | -1040 | 246 | 116 | -4.2 | **0.000048***** |

*^1^Biomarkers with significant differences between the severe and mild groups are made bold.*

*^2^*p-value < 0.05,* ****p-value < 0.001,*
